# AMPK Signaling Regulates Epithelioid Hemangioendothelioma Cell Growth

**DOI:** 10.3390/cancers17172889

**Published:** 2025-09-02

**Authors:** Ryan Kanai, Sarah McMullan, Pukar Baniya, Roselyn S. Dai, Emily Norton, Kaila Lasher, Chloe T. Purello, Caleb N. Seavey, Brian P. Rubin, John M. Lamar

**Affiliations:** 1 Department of Molecular and Cellular Physiology, Albany Medical College, Albany, NY 12208, USA; kanair@amc.edu (R.K.); mcmulls@amc.edu (S.M.); dair@amc.edu (R.S.D.); nortone@amc.edu (E.N.); kaila.lasher99@gmail.com (K.L.); purellot@gmail.com (C.T.P.); 2 Department of Cancer Biology, Lerner Research Institute, Cleveland Clinic Foundation, Cleveland, OH 44195, USA; caseavey@gmail.com (C.N.S.);; 3 Department of General Surgery, Digestive Disease and Surgery Institute, Cleveland Clinic Foundation, Cleveland, OH 44195, USA; 4 Department of Molecular Medicine, Cleveland Clinic Lerner College of Medicine, Case Western Reserve University, Cleveland, OH 44195, USA; 5 Robert J. Tomsich Pathology and Laboratory Medicine Institute, Cleveland Clinic Foundation, Cleveland, OH 44195, USA

**Keywords:** Epithelioid Hemangioendothelioma (EHE), AMPK, TAZ-CAMTA1, YAP, mTOR, Hippo pathway

## Abstract

**Background/Objectives:** Epithelioid Hemangioendothelioma (EHE) is an ultra-rare, metastatic vascular sarcoma with limited therapeutic options. The hallmark of EHE is a chromosomal translocation that produces the *WWTR1-CAMTA1* gene fusion, encoding the aberrant transcriptional regulator TAZ-CAMTA1. Given its central role in the EHE initiation and progression, TAZ-CAMTA1 represents a compelling therapeutic target. **Methods and Results:** In this study, we identified AMP-activated protein kinase (AMPK) as one of several proteins capable of repressing the TAZ-CAMTA1 transcriptional activity in NIH3T3 and HEK293 cell lines. The pharmacologic activation of AMPK inhibited the proliferation of EHE cell lines without inducing apoptosis; however, in contrast to the NIH3T3 and HEK293 models, AMPK activation in EHE cells unexpectedly increased the TAZ-CAMTA1 expression and activity. Notably, elevated TAZ-CAMTA1 expression was also associated with reduced EHE cell growth, suggesting that the induction of TAZ-CAMTA1 may be one mechanism by which AMPK suppresses EHE growth. Additionally, we found that AMPK inhibits mTOR activity and that direct mTOR inhibition also suppresses EHE cell growth. **Conclusions**: Together, these findings demonstrate that AMPK activation impairs EHE viability through dual mechanisms: by promoting TAZ-CAMTA1 expression and by inhibiting mTOR signaling. This work highlights AMPK as a potential therapeutic target in EHE and supports the growing body of evidence favoring mTOR inhibitors as promising treatments for this rare cancer.

## Simple Summary

Epithelioid Hemangioendothelioma (EHE) is an ultra-rare vascular cancer with limited treatment options. It is driven by a genetic mutation that produces a fusion gene encoding a novel fusion protein, TAZ-CAMTA1, making the inhibition of this protein a promising therapeutic strategy. In this study, we discovered that drugs that activate AMP kinase can suppress the proliferation of TAZ-CAMTA1-positive EHE cells. This occurs both through the regulation of TAZ-CAMTA1 and via an independent molecular pathway that can be targeted by an existing FDA-approved drug. Our findings reveal a new therapeutic target that can be exploited to develop more effective treatments for EHE.

## 1. Introduction

Epithelioid Hemangioendothelioma (EHE) is an ultra-rare vascular sarcoma with an incidence of fewer than 1 per 1,000,000 people worldwide [1,2]. EHE can occur at any age but is typically diagnosed in patients in their 20 s and 30 s, with a slightly higher prevalence in women [1,2]. EHE can form in any soft tissue but most commonly occurs in the liver, lungs, and bone [1,2,3] and often presents as a multifocal and metastatic disease. There are no Food and Drug Administration (FDA)-approved treatments for EHE, so patients with an inoperable disease have limited treatment options. Due to its rarity, EHE is severely understudied, and its natural history is poorly understood, which has limited efforts to discover effective treatments. Thus, there is a critical need to advance our understanding of EHE biology so that new treatments can be identified.

EHE is a fusion-driven cancer, with more than 90% of patients positive for a t(1;3)(p36.3;q25) translocation that results in WW Domain-Containing Transcription Regulator Protein 1-Calmodulin-Binding Transcription Activator 1 (*WWTR1-CAMTA1*) gene fusion [4,5]. The majority of the remaining 10% of EHEs are positive for the t(X;11) (p11;q22) translocation that generates a Yes-Associated Protein 1-Transcription factor E3 (*YAP1-TFE3*) gene fusion [6]. The *WWTR1-CAMTA1* fusion gene encodes an oncogenic fusion protein in which the amino terminus of the transcriptional coactivator with a PDZ binding motif (TAZ) protein (encoded by *WWTR1*) is fused to the carboxy terminus of the CAMTA1 protein (encoded by the *CAMTA1* gene). Recent studies have demonstrated that TAZ-CAMTA1 is sufficient to drive EHE [7,8] and is required for tumor maintenance and progression [8]. Interestingly, TAZ-CAMTA1 is unique to EHE, having never been observed in any other cancer. Like endogenous TAZ, the TAZ-CAMTA1 protein is a transcriptional coactivator that promotes tumorigenicity [7,8,9,10,11,12] by partnering with the TEA domain (TEAD) family of transcription factors to regulate gene expression [8,9,10,12]. Indeed, the mutation of Serine 51 to Alanine (S51A), which prevents TAZ’s interaction with the TEADs [13,14,15], eliminated TAZ-CAMTA1’s transcriptional and tumorigenic function [8,9]. Given the critical role of TAZ-CAMTA1 in EHE tumorigenesis, pathways that regulate TAZ-CAMTA1 are attractive therapeutic targets in EHE. However, while several studies have investigated the tumorigenic function of the TAZ-CAMTA1 fusion [7,8,9,10,11,12], little is known about the pathways regulating TAZ-CAMTA1.

While CAMTA1 is relatively understudied, numerous proteins that regulate TAZ and its paralog, Yes-Associated Protein (YAP), have been identified. This includes AMP-activated protein kinase (AMPK), which can regulate YAP and TAZ through multiple mechanisms [16,17,18,19,20]. AMPK is a heterotrimeric protein comprising a catalytic α subunit and β and γ regulatory subunits [21,22]. AMPK acts as a cellular energy sensor that is directly activated by binding to adenosine monophosphate (AMP) in conditions with high amounts of AMP and low amounts of adenosine triphosphate (ATP) [21,22]. In these low-energy conditions, activated AMPK inhibits anabolic pathways to limit energy consumption while promoting catabolic pathways to restore cellular ATP levels [21,22]. Importantly, the AMPK-mediated inhibition of YAP and TAZ has been shown to reduce cancer cell proliferation and re-sensitize cancer cells to anti-cancer drugs [16,17,18,20].

Here, to identify molecular pathways that could be exploited to inhibit EHE cell growth, we performed a focused screen of known regulators of YAP and TAZ in TAZ-CAMTA1-expressing NIH3T3 and HEK293 cells. We found several proteins, including AMPK, that repressed the TAZ-CAMTA1 transcriptional activity. We subsequently found that the pharmacologic activation of AMPK repressed the growth of EHE cells through both TAZ-CAMTA1-dependent and independent mechanisms. Surprisingly, we found that instead of inhibiting TAZ-CAMTA1 as it did in NIH3T3 and HEK293 cells, the activation of AMPK in EHE cells increased TAZ-CAMTA1 levels and activity. We also found that increasing TAZ-CAMTA1 expression repressed EHE cell growth through the inhibition of the mechanistic target of rapamycin (mTOR). Altogether, these findings support AMPK as a potential therapeutic target in EHE.

## 2. Materials and Methods

### 2.1. Cell Lines, Plasmids, and Cloning

All cell lines were cultured at 37 °C and 5% CO_2_ in the appropriate growth medium listed below. Cells were maintained at low passage number and routinely tested for mycoplasma and other bacterial contaminants. Cells were passaged when subconfluent, and all assays were performed on cells at 60–80% confluence. Mouse EHE cells (EHE6, EHE17) [12] used DMEM/F12 supplemented with 20% fetal bovine serum, 0.1 mg/mL Endothelial Cell Growth Supplement (Corning, Corning, NY, USA, Cat#356006), 1X Antibiotic-Antimycotic solution (Millipore Sigma, Burlington, MA, USA, Cat#A5955), and 0.1 mg/mL Heparin (Millipore Sigma, Cat#H3393). HEK293 and 293FT cells used DMEM+ supplemented with 10% fetal bovine serum, 2 mM L-glutamine. NIH3T3 cells used DMEM+ supplemented with 10% bovine calf serum, 2 mM L-glutamine. EHE6 and EHE17 cells were obtained from Dr. Brian Rubin’s lab at the Cleveland Clinic [12]. HEK293 and NIH3T3 cells were purchased from ATCC (Manassas, VA, USA). 293FT cells were purchased from Invitrogen (Carlsbad, CA, USA). Plasmids and their source [9,12,23,24,25,26,27,28,29,30,31,32,33] are listed in Table S1. New vectors were generated using standard cloning procedures, and the source constructs used for each insert and vector backbone are indicated. All newly developed vectors were confirmed by test restriction enzyme cuts and sequenced using Plasmidsaurus to confirm their identity.

### 2.2. Lentivirus and Retrovirus

For virus packaging, 293FT cells were seeded on 6-well plates at 5.0 × 10^5^ cells per well in 2 mL of growth medium. After overnight incubation, cells were transfected (according to the manufacturer’s protocol) with a mixture containing 3.6 μg of DNA (1:1:1 molar ratio of viral vector, packaging vector (Gag/Pol or psPax2) and (VSVG)), 11 μL of X-tremeGENE™9 (Millipore Sigma, Cat#6365779001), and 189 μL of Opti-MEM™ (Thermo Fisher, Waltham, MA, USA, Cat#31985062). The transfection mixture was added to the cells for 24 h and then the mixture was removed, and the cells were fed with fresh DMEM without any supplements. Twenty-four hours after the medium change, the virus-containing supernatant was collected and filtered through a 0.45 μm filter. For stable transduction, cells at roughly 60–80% confluence were incubated with a 1:1 mixture of virus-containing supernatant and fresh growth medium, along with 6 μg/mL of DEAE-Dextran (Millipore Sigma, Cat#D9885). After 24 h, the virus-containing medium was removed, and cells were fed with fresh growth medium. Cells began stable selection with the appropriate antibiotic 72 h after the initial addition of viral supernatant.

### 2.3. TEAD Transcriptional Reporter Assays

Two transcriptional reporter assays were used in this study. Reporter assays in NIH3T3 or HEK293 cells used a transient transfection in which cells were seeded onto 12-well plates in growth medium at 50,000 cells (NIH3T3) or 150,00 cells (HEK293) per well. The next day, using the manufacturer’s protocol, cells were transfected with 800 ng of a 20:1 mixture of TEAD firefly luciferase reporter (pGL3–5xMCAT(SV)-49) [32,33,34], *Renilla* luciferase control plasmid PRL-TK (Promega, Madison, WI, USA), 500 ng of the indicated co-transfected plasmid using 100 μL of Opti-MEM™ (Thermo Fisher, Cat#31985062), 3 μL of Lipofectamine^TM^ 3000, and 2 μL P1000 per 1 μg DNA. Twenty-four hours after transfection, cells were assayed using the Dual-Luciferase Reporter Assay System (Promega, Cat#E1910) and read on a Spectramax I3 plate reader (Molecular Devices, San Jose, CA, USA) as described previously [35]. EHE studies used a stably transduced reporter assay. Here, EHE6 and EHE17 cells were stably transduced with a lentiviral version of the TEAD reporter construct (pLenti-8xGTIIC-Firefly Luciferase-Puro [12]) and a lentiviral *Renilla* luciferase control construct (pLenti-PGK-Blast-Renilla Luciferase [31]. Then, cells were seeded onto 12-well plates at 5.3 × 10^4^ cells per well. Twenty-four hours after seeding, cells were treated with the indicated compound. Twenty-four hours after treatment, cells were assayed using the Dual-Luciferase Reporter Assay System (Promega, Cat#E1910) as detailed above. All TEAD reporter data was normalized by dividing the raw firefly luciferase signal by the raw *Renilla* luciferase signal for each well, averaging replicate wells, and then normalizing this average firefly/*Renilla* value to the average firefly/*Renilla* value of the control sample indicated in the legend.

### 2.4. RNAi

siRNA experiments used Horizon Discovery SMARTPools™ and included a non-targeting control siRNA (Horizon Discovery, Cambridge, UK, Cat#D-001810-01-05) or a SMARTPool™ targeting mouse Yap (Horizon Discovery, Cat#L-046247-01-0005). EHE6 cells were seeded on 6-well plates at 2.9 × 10^5^ cells per well and cultured in growth medium overnight. The next day, cells were changed into growth medium without heparin or serum and transfected with a mixture containing 70 pmol of siRNA, 9 μL of Lipofectamine™ RNAiMAX (Thermo Fisher, Cat#13778150), and 230 μL Opti-MEM™ (Thermo Fisher, Cat#31985062) according to the manufacturer’s protocol. Twenty-four hours after the transfection, cells were fed with growth medium. Twenty-four hours later, the cells were trypsinized and seeded for Western blot, qPCR, or cell growth assays.

### 2.5. Western Blot

Cells were seeded on 10 cm plates at 6.8 × 10^5^ cells per plate in growth medium. The next day, cells were treated with drug for 24 h then lysed in Cell Lysis Buffer (Cell Signaling Technology, Danvers, MA, USA, Cat#9803) containing Pierce™ (Rockford, IL, USA) Protease and Phosphatase Inhibitor Mini Tablets (Thermo Fisher, Cat#A32959). The protein concentration was determined using the Pierce™ BCA protein assay kit (Thermo Fisher, Cat#23225), and equal protein (10–20 ug) was subjected to 10% SDS-PAGE, transferred to PVDF membranes, and assayed by Western blot. Primary antibody information is provided in Table S2. Blots were quantified using Image Lab Software (Bio-Rad, Hercules, CA, USA Version 6.0.1) in which band intensities were quantified and subsequently normalized to the housekeeping gene used for that experiment (either GAPDH or Beta-actin). Housekeeping-normalized phosphorylated protein bands were then normalized to housekeeping-normalized total protein bands.

### 2.6. qPCR

Cells were seeded on 60 mm plates at 3.75 × 10^5^ cells per plate in growth medium. The next day, cells were treated with drug for 24 h and then lysed in TRIzol^®^ (Thermo Fisher, Cat#15596018). RNA was isolated using a PureLink^TM^ RNA mini kit (Thermo Fisher, Cat#12183025), and 200 ng of total RNA was reverse transcribed to produce cDNA template using the qScript cDNA synthesis kit (QuantaBio, Beverly, MA, USA, Cat#95048). qPCR was carried out using 2 μL of cDNA, 10 μL PerfeCTa SYBR Green Fastmix Universal (QuantaBio, Cat#101414-270), and 2 pmol of each primer (See Table S3). The reaction mixture was brought to a total volume of 20 μL with nuclease-free water. qPCR reactions were run using a CFX Connect real-time PCR detection system (Bio-Rad, Cat#1855201) according to the manufacturer’s instructions. PCR conditions were 95 °C for 30 s, followed by 40 cycles of 95 °C for 10 s and 55.9 °C for 30 s, followed by a melt temperature analysis. The CFX Maestro software (Bio-Rad, Version 3.1.1517.0823) was used to calculate the fold-change in mRNA for each indicated gene for each sample relative to a pre-determined control sample using the ΔΔCt method and *Gapdh* as a reference gene.

### 2.7. Cell Viability and Growth

Both cell viability and cell number were assayed from the same well of cells as follows. First, cells were seeded at 3200 cells per well on 96-well black, clear bottom plates (Millipore Sigma, Cat#BR781971). Twenty-four hours later cells were treated with the indicated drug for 96 h and then assayed using a CCK8 live cell counting kit (Dojindo, Rockville, MD, USA, Cat#CK-04) according to the manufacturer’s protocol. After the CCK8 assay was completed, cells were fixed with 4% paraformaldehyde and stained with either 10 μg/mL Hoechst stain in 1X PBS (Thermo Fisher, Cat#H1399) or 1X NucSpot Live 488 (Biotium, Fremont, CA, USA, Cat#40081) in 1X PBS for 60 or 30 min at 37 °C, respectively. Then, nuclei were counted using a Cytation 5 Cell Imaging Multimode Reader (BioTek, Shoreline, WA, USA). For multi-day CCK8 dose curves, absorbance values from 4 replicate wells were averaged for each sample at each timepoint. Then, the average absorbance for each sample at every timepoint was normalized to the average of the untreated wells at 24 h. For all other CCK8 assays and nuclei counting, absorbance values or cell numbers from 4 replicate wells at the 96 h timepoint were averaged for each sample. Then, the sample averages were normalized to the untreated well at the 96 h timepoint.

### 2.8. Immunocytochemistry/Immunofluorescence (ICC/IF)

To assess changes in the number of Ki67^+^ cells, EHE cells were seeded at 3200 cells per well in 96-well black, clear bottom plates (Millipore Sigma, Cat#BR781971). Twenty-four hours later, cells were treated with the indicated drug for 96 h, fixed with 4% paraformaldehyde, and stained with Ki67 antibody (see Table S2) and 0.5 μg/mL DAPI (Millipore Sigma, Cat#D9542) in DMSO. Automated counting of total cells (based on counting DAPI-stained nuclei) and Ki67^+^ cells were performed using a Cytation 5 Cell Imaging Multimode Reader (Biotek).

### 2.9. β-galactosidase Senescence Assay

A SPiDER-βGal kit (Dojindo, Cat#SG05) was used according to manufacturer instructions to assess changes in cellular senescence. Briefly, cells were seeded at 3200 cells per well in 96-well black, clear bottom plates (Millipore Sigma, Cat#BR781971). Twenty-four hours later, cells were treated daily with the indicated drug for 96 h. Then, live cells were stained with 10 μg/mL Hoechst stain in 1X PBS and total Hoescht fluorescence was measured using the fluorometer function on a Cytation 5 Cell Imaging Multimode Reader (BioTek). Then, cells were lysed, and β-galactosidase was stained following the SPiDER-βGal kit (Dojindo, Cat#SG05) instructions, and total β-galactosidase fluorescence was measured using the fluorometer function on the Cytation 5 Cell Imaging Multimode Reader. Total β-galactosidase fluorescent signal was normalized to total Hoescht fluorescet signal to account for differences in cell number between wells.

### 2.10. Statistical Analysis

Statistical analyses were performed in GraphPad (GraphPad, San Diego, CA, USA) Prism version 10.1.1). Detailed descriptions of the statistical tests used to determine significance, the number of ns, and data normalization are indicated in the Figure Legends. Briefly, for experiments in which two samples were compared, statistical significance was determined using either unpaired *t*-test or single sample *t*-test in which the indicated control sample was set to 1. Experiments comparing more than 2 samples used One-Way ANOVA with Dunnett’s post hoc test. In the figures, statistical significance is indicated with the following: * *p* ≤ 0.05; ** *p* ≤ 0.01; *** *p* ≤ 0.001; **** *p* ≤ 0.0001; and n.s. *p* > 0.05. All scatter plots show mean ± SD unless noted otherwise in the legend.

## 3. Results

### 3.1. Identification of Negative Regulators of TAZ-CAMTA1-TEAD Transcriptional Activity

**Figure 1:**
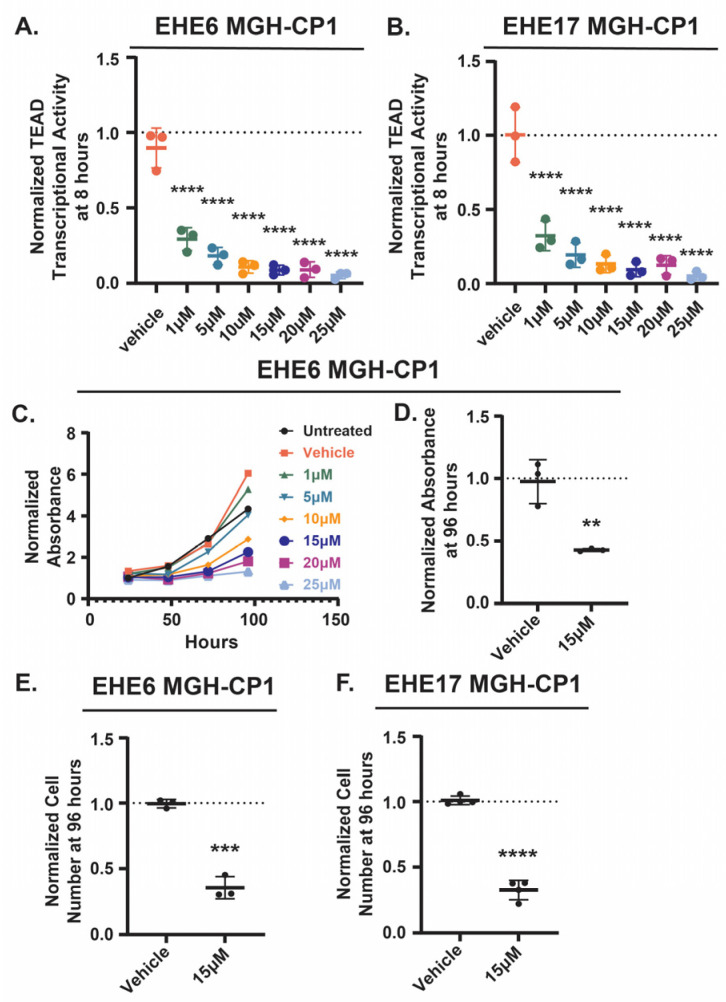
EHE cells are sensitive to TEAD inhibition. EHE6 (**A**,**C**–**E**) and EHE17 (**B**,**F**) cells were treated with the indicated doses of the TEAD inhibitor MGH-CP1 for 8 h (**A**,**B**) or every day for 4 days (**C**–**F**) and then assayed for TEAD transcriptional activity (**A**,**B**) or for cell viability using both a CCK8 assay (**C**,**D**) and by counting cell numbers (**E**,**F**). (**A**,**B**,**D**–**F**). The plots show the mean ± SD, and each group was normalized to untreated cells (represented by the dotted line at Y = 1). (**A**,**B**) *n* = 3 independent experiments where 2 wells were read in duplicate and were averaged; **** *p* ≤ 0.0001 by One-Way ANOVA with Dunnett’s post hoc test comparing each group to the vehicle control. (**C**) For each group, the absorbance at each timepoint was normalized to the absorbance of untreated cells at the 24 h timepoint for *n* = 1 experiment where 4 wells were averaged. (**D**–**F**) *n* = 3 (**D**,**E**) or *n* = 4 (**F**) independent experiments where 4 replicate wells were averaged; ** *p* ≤ 0.01, *** *p* ≤ 0.001, and **** *p* ≤ 0.0001 by unpaired *t*-test.

Previous findings have demonstrated that TAZ-CAMTA1 is critical for EHE development and tumor progression [7,8] and that interaction with TEAD transcription factors is required for TAZ-CAMTA1’s tumorigenic function [9]. This suggests that pathways that regulate TAZ-CAMTA1-TEAD activity could be exploited to inhibit EHE growth. To confirm that EHE cell growth is sensitive to TEAD inhibition we treated two murine EHE cell lines (EHE6 and EHE17 [12]) with the TEAD inhibitor, MGH-CP1 [36], at a range of doses similar to those previously reported [12,36,37]. We then assayed TEAD transcriptional activity using a lentiviral TEAD reporter construct [12] and cell growth using both the CCK8 cell viability assay and by counting cells. As expected, the MGH-CP1 treatment reduced TEAD transcriptional activity in both cell lines in a dose-dependent manner at 8 (Figure 1A,B) and 24 h (Figure S1A,B). EHE cell viability was also reduced in a dose-dependent manner (Figure 1C and Figure S1C). Indeed, there was a significant reduction in both the cell viability and cell number in both EHE cell lines at 15 μM MGH-CP1, a dose which showed a significant reduction in TEAD transcriptional activity (Figure 1D–F and Figure S1D). Altogether, these data confirm that the growth of these EHE cells is dependent upon the TAZ-CAMTA1-TEAD function, suggesting that pathways that repress TAZ-CAMTA1 could be exploited to inhibit EHE growth.

EHE6 and EHE17 cell lines cannot be efficiently transfected with cDNA constructs, making it difficult to test candidate TAZ-CAMTA1 regulators using these cells. Therefore, we used HEK293 and NIH3T3 cells ectopically expressing TAZ-CAMTA1, which are two more genetically tractable models used to study TAZ-CAMTA1 function [9,10,11]. We first infected NIH3T3 cells with several concentrations of a retrovirus encoding TAZ-CAMTA1 and confirmed that the TEAD transcriptional activity increased as the TAZ-CAMTA1 protein expression increased (Figure S2A–C). The stable expression of TAZ-CAMTA1 also induced TEAD transcriptional activity in HEK293 cells, and this was dependent upon interactions with TEADs because a mutant form of TAZ-CAMTA1 unable to bind TEADs (TC-S51A) did not induce TEAD transcriptional activity (Figure S2D,E). This confirms that our TEAD reporter assay can be used to measure the TAZ-CAMTA1-TEAD transcriptional activity.

The TAZ-CAMTA1 fusion retains several residues and domains through which other pathways negatively regulate TAZ and/or its paralog, YAP1 [18,20,38,39,40,41,42,43,44] (Figure S3A,B). Therefore, we hypothesized that TAZ-CAMTA1 would be sensitive to negative regulation by several proteins that regulate TAZ-CAMTA1 through this part of the protein. NIH3T3 cells stably expressing TAZ-CAMTA1 were co-transfected with the TEAD reporter constructs and either an empty vector (EV), AMPKα1 [28], AMPKα2 [29], Protein Tyrosine Phosphatase Non-Receptor Type 14 (PTPN14) [26], Gelsolin [27], Cofilin [30], an activated form of Cofilin (S9A) [30], an activated from of Glycogen Synthase Kinase-3 beta (GSK3β) [23], an activated form of Angiomotin (P130) [24], or two distinct NF2 isoforms and then were assayed for TEAD reporter activity. This revealed several proteins able to repress TAZ-CAMTA1-TEAD transcriptional activity (Figure S3C). We retested putative proteins in HEK293 cells expressing TAZ-CAMTA1 and found that they also repressed TAZ-CAMTA1-TEAD (Figure S3D). Thus, TAZ-CAMTA1 remains sensitive to regulation by pathways that repress TAZ through its N-terminus.

### 3.2. AMPK Activation Represses EHE Cell Growth

**Figure 2:**
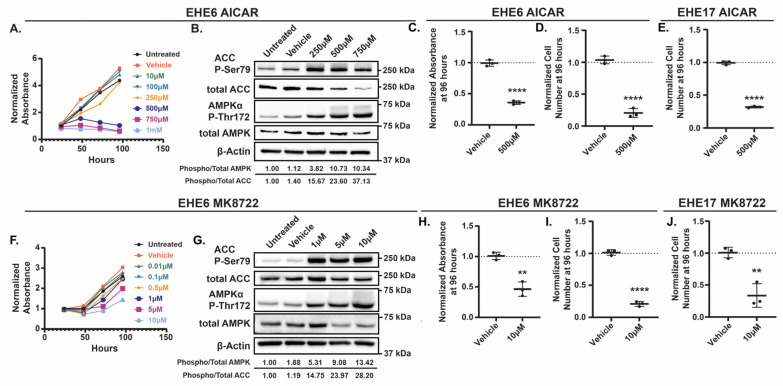
The AMPK activators AICAR and MK8722 inhibit EHE cell viability. EHE6 (**A**–**D**,**F**–**I**) and EHE17 (**E**,**J**) cells were treated with the AMPK activators AICAR (**A**–**E**) or MK8722 (**F**–**J**) at the indicated doses every day for 4 days (**A**,**C**–**F**,**H**–**J**) or for 24 h (**B**,**G**). Cells were then assayed for cell viability using a CCK8 assay (**A**,**C**,**F**,**H**) and by counting cell numbers (**D**,**E**,**I**,**J**) or by Western blot (**B**,**G**). (**A**,**F**) For each group, the absorbance at each timepoint was normalized to the absorbance of untreated cells at the 24 h timepoint; *n* = 1 independent experiment where 4 wells were averaged. (**B**,**G**) Representative Western blots with quantification of phosphorylated over total AMPK and ACC are shown. (**C**–**E**,**H**–**J**) The plots show the mean ± SD, and each group was normalized to untreated cells (represented by dotted lines at Y = 1 on graphs). *n* = 3 independent experiments where 4 replicate wells were averaged; ** *p* ≤ 0.01 and **** *p* ≤ 0.0001 by unpaired *t*-test.

Among the regulators of TAZ-CAMTA1 that we identified, AMPK was particularly interesting because therapeutic AMPK activators are available. To test if AMPK activation can repress EHE cell growth, we treated EHE6 and EHE17 cells with the AMP-mimetic 5-Aminoimidazole-4-carboxyamide ribonucleoside (AICAR) [45,46] or the small-molecule MK8722 [47,48], two direct pharmacologic activators of AMPK. AICAR and MK8722 each reduced the viability of both EHE cell lines in a dose-dependent manner (Figure 2A,F and Figure S4A,D). To confirm that the doses of AICAR and MK8722 that repressed EHE viability correlated with increased AMPK activation, we performed Western blots for the phosphorylation of the α subunit of AMPK at Threonine 172 (Thr172) [49,50,51] and the phosphorylation of the AMPK substrate, Acetyl CoA Carboxylase (ACC), at Serine 79 (Ser79) [52,53], which are two established readouts of AMPK activity. MK8722 and AICAR each increased the AMPKα and ACC phosphorylation in both EHE cell lines (Figure 2B,G and Figure S4B,E). Although both drugs started to induce AMPKα and ACC phosphorylation at doses that did not inhibit EHE viability, robust AMPK activation occurred at 10 μM of MK8722 and 500 μM of AICAR, doses that did reduce EHE cell viability (Figure 2A,F and Figure S4A,D). These doses, which are similar to those used in other studies with AICAR [54,55,56] or MK8722 [48,57,58], substantially reduced the cell viability and cell number in both EHE cell lines (Figure 2C–E,H–J and Figure S4C,F).

**Figure 3:**
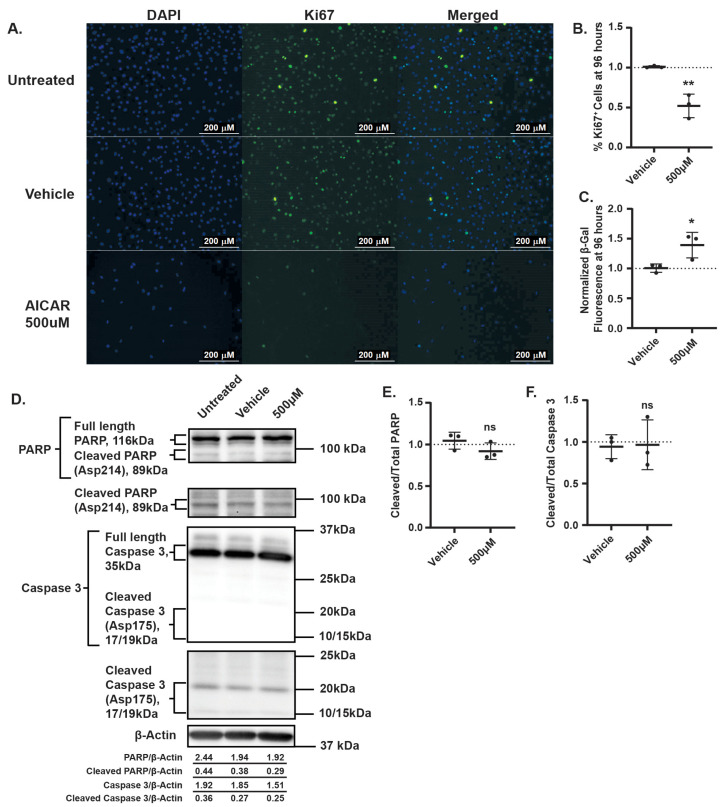
AICAR reduces EHE cell proliferation but does not induce apoptosis. EHE6 cells were treated with AICAR for 96 h and assayed by immunofluorescence for Ki67 (**A**,**B**) or by a SPiDER-βGal kit for β-Galactosidase (β-Gal) (**C**). (**A**) Representative fields of view showing Dapi (blue)- and Ki67 (green)-positive nuclei with quantification in (**B**). (**D**–**F**) EHE6 cells were treated with AICAR for 24 h and assayed by Western blot for changes in total and cleaved Caspase-3 and PARP. (**D**) Representative images of Western blots with quantification of the displayed blots. (**E**,**F**) Band intensity was quantified and normalized to β-actin. For all graphs, data represent mean ± SD, and each group was normalized to untreated cells (represented by dotted lines at Y = 1 on graphs). (**A**–**C**) *n* = 3 independent experiments where 2 (**A**–**B**) or 4 (**C**) replicate wells were averaged; ** *p* ≤ 0.01 and * *p* ≤ 0.05 by unpaired *t*-test. (**E**,**F**) *n* = 3 independent experiments; ns = not significant by unpaired *t*-test.

We next tested whether AMPK activation represses proliferation, induces apoptosis, or promotes senescence. The treatment of EHE6 cells with either AICAR (Figure 3A,B) or MK8722 (Figure S5A,B) substantially reduced the percentage of Ki67-positive cells. Although both the AICAR and MK8722 treatment also increased the percentage of β-Galactosidase (β-Gal)-positive cells (Figure 3C and Figure S5C), this increase was only statistically significant in the AICAR-treated cells. Western blot analysis showed that neither the AICAR nor MK8722 treatment altered the levels of cleaved Caspase-3 or cleaved PARP, indicating that AMPK activation does not induce apoptosis in EHE cells (Figure 3D–F and Figure S5D–F). Collectively, these results show that the robust pharmacological activation of AMPK leads to reduced EHE cell growth predominantly by repressing cell proliferation.

### 3.3. AMPK Activation Represses EHE Through Two Distinct Mechanisms

**Figure 4:**
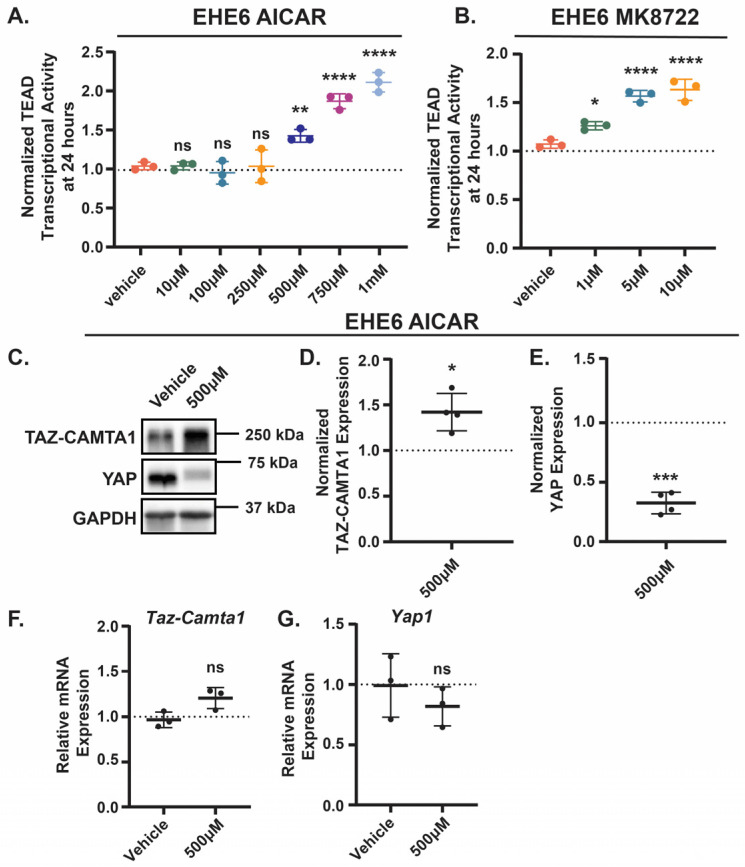
AMPK activation increases TAZ-CAMTA1 and decreases YAP levels. EHE6 cells were treated for 24 h with AICAR (**A**,**C**–**G**) or MK8722 (**B**) and assayed for TEAD transcriptional activity (**A**,**B**) by Western blot (**C**–**E**) or by qPCR (**F**,**G**). All plots show the mean ± SD. (**A**,**B**) Each group was normalized to untreated cells (represented by dotted lines at Y = 1 on graphs), *n* = 3 independent experiments where 2 wells read in duplicate were averaged; ns = not significant, * *p* ≤ 0.05, ** *p* ≤ 0.01, and **** *p* ≤ 0.0001 by One-Way ANOVA with Dunnett’s post hoc test comparing each group to vehicle-treated cells. (**C**) Representative Western blots with band intensity quantified and normalized to GAPDH (**D**,**E**). *n* = 4 independent Western blot experiments where each group was normalized to vehicle-treated cells (represented by dotted lines at Y = 1 on graphs); * *p* ≤ 0.05 and *** *p* ≤ 0.001 by one sample *t*-test. (**F**–**G**) *n* = 3 independent experiments where 3 technical replicates were averaged, and each group was normalized to untreated cells (represented by dotted lines at Y = 1 on graphs); ns = not significant by unpaired *t*-test.

Given that AMPK repressed TAZ-CAMTA1 in NIH3T3 and HEK293 cells, we next investigated whether AMPK activation represses EHE cell growth by inhibiting TAZ-CAMTA1. Paradoxically, instead of decreasing TAZ-CAMTA1-TEAD transcriptional activity, the treatment with either AICAR or MK8722 resulted in a dose-dependent increase in activity in both cell lines (Figure 4A,B and Figure S6A,B). Interestingly, AMPK activation increased TAZ-CAMTA1 protein levels (Figure 4C,D and Figure S6C,D), which is a potential explanation for this increased TEAD transcriptional activity. Both drugs also decreased YAP levels in these cells (Figure 4C,E and Figure S6C,E). We did not observe any change in the expression of TAZ-CAMTA1 or YAP at the mRNA level (Figure 4F,G and Figure S6F,G), suggesting that in EHE cells, AMPK regulates TAZ-CAMTA1 and YAP expression at the post-translational level. Thus, despite our initial observation that AMPK can repress ectopically expressed TAZ-CAMTA1 in NIH3T3 and HEK293 cells, it appears to promote TAZ-CAMTA1 protein expression and TEAD transcriptional activity in EHE cells. The fact that TAZ-CAMTA1 is regulated differently in EHE cells than it is in other cell lines highlights the importance of the use EHE models to study the regulation of this oncogene and test drugs that inhibit its function.

**Figure 5:**
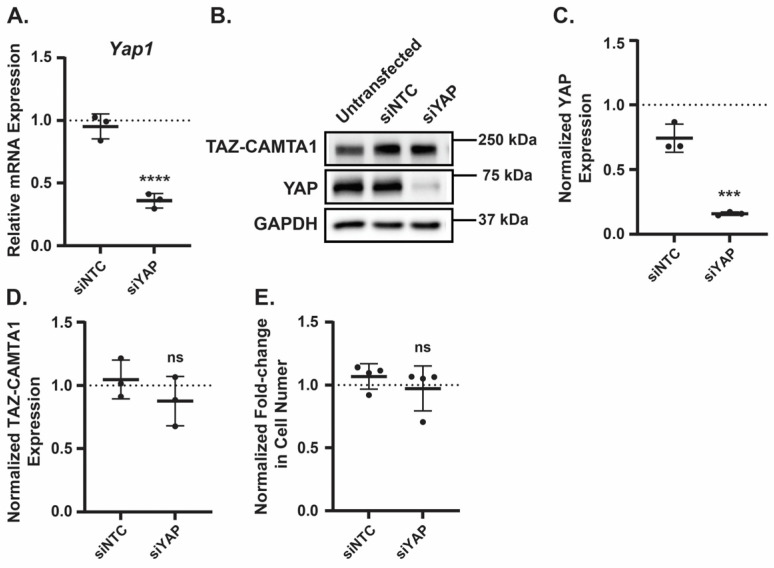
Loss of YAP does not affect EHE cell viability or TAZ-CAMTA1 expression. (**A**–**E**) EHE6 cells were transfected with a non-targeting siRNA (siNTC) or pooled siRNAs that target YAP (siYAP) and then assayed by qPCR (**A**), Western blot (**B**–**D**), or for cell viability by counting cell number (**E**). All plots show the mean ± SD where each group was normalized to untransfected cells (represented by dotted lines at Y = 1 on graphs). (**B**) Representative Western blots with band intensity were quantified and normalized to GAPDH for *n* = 3 independent Western blot experiments (**C**,**D**). *n* = 3 (**A**) or *n* = 4 (**E**) independent experiments where 4 replicates were averaged; ns = not significant, *** *p* ≤ 0.001 and **** *p* ≤ 0.0001 by unpaired *t*-test.

The results in Figure 4 were unexpected given that TAZ-CAMTA1 is known to play a causal role in EHE [7,8] and that we and others have found that the inhibition of TAZ-CAMTA1-TEAD activity represses EHE cell growth (Figure 1 and [12]). However, recent work revealed that TAZ-CAMTA1 can cause oncogene-induced senescence when expressed at high levels in endothelial progenitor cells [59]. This raises the intriguing possibility that while necessary for EHE cell growth, TAZ-CAMTA1 can also be detrimental if its activity is too high. YAP is also an established driver of cell growth in many cancers [60,61], and it can partner with other transcription factors in addition to TEADs. Thus, we next tested if the decrease in EHE cell viability caused by AMPK activation is due to the changes in either TAZ-CAMTA1 or YAP levels. To determine whether reduced YAP expression impairs EHE cell growth, we transfected EHE6 cells with either a non-targeting control siRNA (siNTC) or pooled siRNAs targeting YAP and then confirmed YAP knockdown by a qPCR and Western blot (Figure 5A–C). EHE cell growth was not impaired by the loss of the YAP expression (Figure 5E), indicating that the AMPK-mediated decrease in YAP expression is not responsible for the reduced EHE cell viability. In addition, we did not observe any changes in TAZ-CAMTA1 protein expression in the YAP knockdown cells (Figure 5D), indicating that the increase in TAZ-CAMTA1 expression caused by the AMPK activation is not due to the loss of YAP levels.

**Figure 6:**
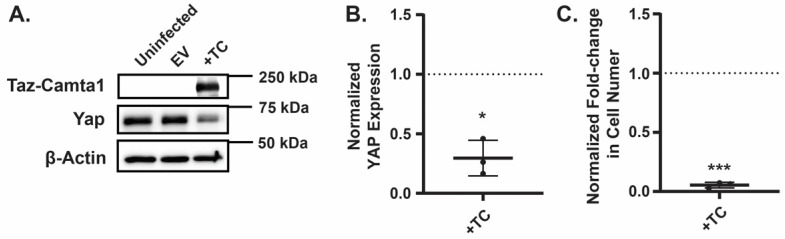
Increased TAZ-CAMTA1 expression reduces EHE cell viability and YAP expression. (**A**,**B**) EHE6 cells were stably transduced with control empty vector (EV) or TAZ-CAMTA1 and then assayed by Western blot (**A**,**B**) or for cell viability by counting cell number (**C**). All plots show the mean ± SD where each group was normalized to cells transduced with EV (represented by dotted lines at Y = 1 on graphs) (**A**) Representative Western blots. (**B**) Band intensity was quantified and normalized to GAPDH for *n* = 3 independent experiments. (**C**) *n* = 3 independent experiments where 4 replicate wells were averaged. * *p* ≤ 0.05 and *** *p* ≤ 0.001 by one sample *t*-test.

To investigate whether elevated TAZ-CAMTA1 expression directly impairs EHE cell growth, we generated EHE6 cells stably expressing either a control empty vector (EV) or exogenous TAZ-CAMTA1. The Western blot analysis confirmed that TAZ-CAMTA1 levels were significantly higher in the engineered cells compared to controls (Figure 6A). Strikingly, cells with increased TAZ-CAMTA1 expression exhibited reduced cell growth (Figure 6C), supporting the idea that excessive TAZ-CAMTA1 activity can be detrimental to EHE cells. Interestingly, we also observed a decrease in YAP expression in cells overexpressing TAZ-CAMTA1 (Figure 6A,B), although this reduction was not as pronounced as the decrease following the AMPK activation. Together, these findings suggest that AMPK activation leads to an upregulation of TAZ-CAMTA1, representing a potential mechanism through which AMPK exerts its growth-inhibitory effects in EHE.

**Figure 7:**
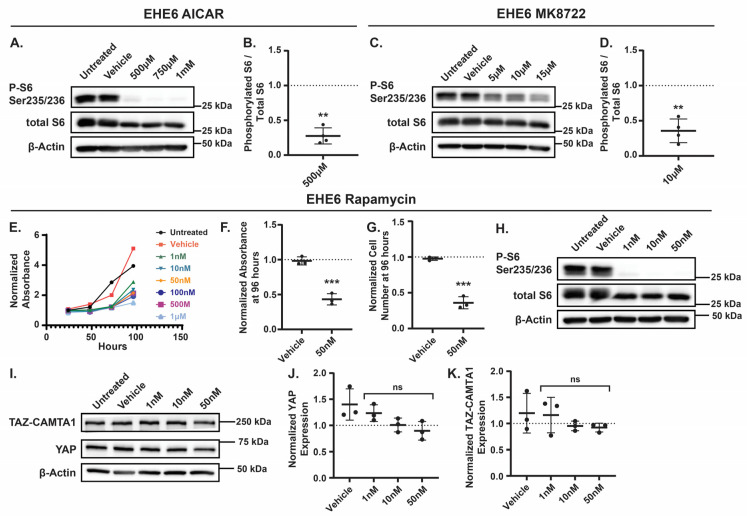
AMPK activation represses mTOR activity, inhibiting EHE cell viability. EHE6 cells were treated for 24 h with AICAR (**A**,**B**) or MK8722 (**C**,**D**) and assayed by Western blot. (**E**–**K**) EHE6 cells were treated with the mTOR inhibitor rapamycin at the indicated doses every day for 4 days (**E**–**G**) or for 24 h (**H**–**K**). Cells were then assayed for cell viability using a CCK8 assay (**E**,**F**) and by counting cell number (**G**) or by Western blot (**H**–**K**). (**A**,**C**,**H**,**I**) Representative Western blots. (**B**,**D**) Band intensity was quantified and normalized to GAPDH for *n* = 4 independent experiments, and the plots show the mean ± SD where each group was normalized to vehicle-treated cells (represented by dotted lines at Y = 1 on graphs); ** *p* ≤ 0.01 by one sample *t*-test. (**E**) Each group was normalized to the absorbance of untreated cells at the 24 h timepoint; *n* = 1 independent experiment where 4 wells were averaged. (**F**,**G**,**J**,**K**) The plots show the mean ± SD, and each group was normalized to untreated cells (represented by dotted lines at Y = 1 on graphs). (**F**,**G**) *n* = 3 independent experiments where 4 replicate wells were averaged; *** *p* ≤ 0.001 by unpaired *t*-test. (**J**,**K**) *n* = 3 independent experiments; ns = not significant by One-Way ANOVA with Dunnett’s post hoc test.

Although our data suggests that the AMPK-mediated increase in TAZ-CAMTA1 expression contributes to the inhibition of EHE cell growth, AMPK is an established inhibitor of mTOR [62,63,64,65,66], and growing pre-clinical and clinical data demonstrates that the mTOR inhibitor rapamycin (also called Sirolimus) is a viable treatment for EHE [67,68,69]. Therefore, we tested if AMPK activation in EHE cells inhibits mTOR activity and, if so, whether this impairs EHE cell growth. First, we treated EHE6 cells with doses of AICAR or MK8722 that repressed EHE growth and examined mTOR activity by measuring the phosphorylation of the S6 ribosomal protein at Ser235/236, a direct target of mTOR [70,71]. As expected, we observed a decrease in S6 phosphorylation with either AICAR or MK8722 treatment (Figure 7A–D), indicating that AMPK activation inhibits mTOR in EHE cells. To test if mTOR inhibition represses EHE cell growth, we treated EHE6 and EHE17 cells with the mTOR inhibitor, rapamycin, and observed a significant inhibition of cell growth at all doses we tested (Figure 7E and Figure S7A). Indeed, 50 nM rapamycin treatment inhibited cell viability and cell number by greater than 50% in both EHE6 and EHE17 cells (Figure 7E–G and Figure S7A–C). Western blots confirmed that the mTOR activity (S6 phosphorylation) was significantly impaired at this dose of rapamycin (Figure 7H and Figure S7D). Rapamycin treatment did not result in any changes in either YAP or TAZ-CAMTA1 protein levels in EHE cells (Figure 7I–K). Altogether, our results suggest that AMPK activation impairs EHE cell growth through both the inhibition of mTOR signaling and independently by increasing TAZ-CAMTA1 protein expression.

## 4. Discussion

Given the lack of FDA-approved treatments for EHE, the goal of this study was to identify new therapeutic targets that could be exploited to inhibit EHE cell growth. We identified AMPK signaling as a promising avenue, as its activation not only suppresses mTOR signaling, but also unexpectedly increases TAZ-CAMTA1 expression, ultimately leading to reduced EHE cell growth.

### 4.1. AMPK Regulates EHE Cell Growth Through Inhibition of mTOR

AMPK is an established regulator of mTOR [62,63,64,72] and can inhibit mTOR signaling through multiple mechanisms. It can directly phosphorylate the GTPase-activating protein (GAP) Tuberous Sclerosis 2 Protein (TSC2) on Tyrosine 1227 and Serine 1345, leading to the activation of the TSC1/2 complex [64,66]. This allows TSC1/2 to interact with Ras Homolog Enriched In Brain (Rheb) and promote the hydrolysis of guanosine triphosphate (GTP) to guanosine diphosphate (GDP) [73,74,75,76,77], ultimately reducing mTOR activity [71,78,79]. AMPK can also phosphorylate Raptor, a scaffold for proteins subject to mTOR phosphorylation [80,81,82], which in turn inhibits mTORC1 signaling [65]. We found that AMPK represses mTOR signaling in EHE cells and that mTOR inhibition impairs cell growth (Figure 7). This is consistent with a recent study that used an EHE patient-derived xenograft (PDX) and corresponding cell line to show that Sirolimus suppressed the growth of human EHE cells in vitro and repressed EHE growth in vivo [67]. Although Sirolimus has not yet received FDA or European Medicines Agency approval for EHE, it is used off-label to treat EHE and has shown efficacy in some patients [1,67,68,69,83,84,85,86,87,88,89,90,91,92]. Thus, our findings further support the potential of Sirolimus in EHE and suggest that the pharmacologic activation of AMPK may provide an additional way to target mTOR in EHE patients.

mTOR plays critical roles in cell metabolism, growth, proliferation, and survival [93,94,95,96], and mTOR inhibition impairs tumor cell proliferation by repressing metabolic pathways critical for protein and lipid biosynthesis [94,97,98]. What makes EHE cells susceptible to mTOR inhibition is unclear. Cancers with elevated mTOR signaling are often more susceptible to mTOR inhibition [99,100], and EHE cells appear to have elevated PI3K-AKT-mTOR signaling [7,8,101]. Whether TAZ-CAMTA1 itself plays a role in EHE cell susceptibility to mTOR inhibition has not been explored. Although mTOR can promote YAP/TAZ expression by inhibiting autophagy [102], our results suggest that mTOR inhibition does not alter YAP or TAZ-CAMTA1 expression in EHE cells. However, there is also evidence that YAP and TAZ can promote mTOR activity [15,103,104,105], raising the possibility that mTOR activation is a downstream consequence of TAZ-CAMTA1 in EHE. Alternatively, sensitivity to mTOR inhibition could be due to the endothelial origin of EHE, as other vascular sarcomas and vascular malformations have elevated mTOR signaling [83,106,107,108,109,110] and show sensitivity to Sirolimus [91,111,112,113,114,115,116,117,118]. It is also possible that in EHE cells, basal AMPK signaling is too low to effectively restrain mTOR signaling. Understanding the causes of the elevated mTOR activity in EHE cells could help explain why not all EHE patients benefit from Sirolimus and provide a more effective way to identify the ones that will.

### 4.2. AMPK Regulation of YAP and TAZ-CAMTA1 in EHE

AMPK can inhibit YAP functions through multiple mechanisms, including the activation of the Large Tumor Suppressor Kinase (LATS) [16,18,20], as well as direct phosphorylation at Serine 61 and Serine 94 [18,19], with the latter preventing YAP-TEAD interactions [13,14]. Based on these data and given the paralogous nature of YAP and TAZ, we hypothesized that AMPK would inhibit TAZ-CAMTA1. While the AMPK activation in NIH3T3 and HEK293 cells did repress TAZ-CAMTA1-TEAD transcriptional activity (Figure S3), this was not the case in EHE cells. Instead, TAZ-CAMTA1 protein levels and TEAD transcriptional activity both increased following the AMPK activation (Figure 4). We further showed that increasing the TAZ-CAMTA1 protein expression inhibits EHE cell growth (Figure 6), suggesting that AMPK represses EHE growth in part through the upregulation of TAZ-CAMTA1. An important caveat here is that the exogenous expression of TAZ-CAMTA1 increased its protein levels to a much higher extent than the AMPK activation. Despite trying a range of viral supernatants, we could not generate EHE cells that showed TAZ-CAMTA1 levels comparable to those in which we pharmacologically activated AMPK. Thus, it is difficult to determine how significantly the AMPK-mediated induction of TAZ-CAMTA1 impacts cell growth. Nevertheless, our findings are consistent with a recent study that showed that endothelial progenitor cells with higher TAZ-CAMTA1 expression grew slower than cells with lower TAZ-CAMTA1 expression [59]. This study further demonstrated that high TAZ-CAMTA1 expression caused senescence by driving hypertranscription [59]. Elevated YAP or TAZ activity can also cause hypertranscription in other cells [119]. We did observe increased senescence in EHE cells following AMPK activation by both AICAR and MK8722, though the latter did not reach statistical significance (Figure S5). Both drugs reduced the number of Ki67-positive cells in a statistically significant manner, but this inhibition of proliferation was less dramatic in MK8722-treated cells. This seems to suggest that AICAR is more potent in these cells than MK8772. Consistently, the increase in TAZ-CAMTA1 expression was also not as dramatic with MK8722 as it was with AICAR. Collectively, these data suggest that while the reduction in proliferation caused by AMPK-mediated repression of mTOR is largely responsible for the inhibition of the EHE cell growth that we observed, the induction of TAZ-CAMTA1 may also contribute by driving senescence in a subset of the EHE cells. This observation is interesting as it suggests that too much TAZ-CAMTA1-TEAD activity may slow the growth of EHE cells. However, it is also clear that the loss of TAZ-CAMTA1 or the inhibition of TAZ-CAMTA1-TEAD activity is detrimental to EHE cells (Figure 1 and [12,120]). Thus, these findings suggest that the level of TAZ-CAMTA1 activity needs to be tightly controlled, which could have important therapeutic implications given that TEAD inhibitors are currently being tested in EHE patients.

The mechanism by which AMPK increases TAZ-CAMTA1 levels remains unclear. Given that YAP levels decreased concurrently with increased TAZ-CAMTA1, we initially hypothesized that AMPK was causing the degradation of YAP, which led to an increase in TAZ-CAMTA1. However, the direct knockdown of YAP did not alter the TAZ-CAMTA1 expression. Instead, we found that the overexpression of TAZ-CAMTA1 reduced YAP expression, indicating that the AMPK-mediated increase in TAZ-CAMTA1 expression is partially responsible for the decreased YAP levels we observed. This did not play a causal role in the AMPK-mediated repression of EHE cell growth, suggesting that YAP is not essential for EHE cell survival or proliferation. We also ruled out a role for the AMPK-mediated suppression of mTOR in regulating TAZ-CAMTA1 levels (Figure 7).

AMPK activation did not increase TAZ-CAMTA1 mRNA expression (Figure 4), indicating it is either enhancing its translation or preventing its degradation. The TAZ portion of the TAZ-CAMTA1 fusion retains the N-terminal phosphodegron, which when phosphorylated by GSK3β leads to the proteasomal degradation of TAZ through β-TRCP [42]. Our data in NIH3T3 and HEK293 cells suggests that TAZ-CAMTA1 is still sensitive to this regulation, since the overexpression of GSK3β inhibited TAZ-CAMTA1-TEAD activity (Figure S3). Importantly, AMPK can regulate proteasomal degradation by influencing the activity and expression of ubiquitin ligases (reviewed in [121]) and by suppressing the proteasome itself [122,123,124]. This raises the possibility that AMPK increases TAZ-CAMTA1 protein levels by inhibiting its proteasomal degradation.

### 4.3. Negative Regulation of TAZ-CAMTA1 Is Context-Dependent

Since TAZ-CAMTA1 plays a causal role in EHE [7,8] and TAZ-CAMTA1-TEAD activity is required for EHE cell growth [12,120], we predicted that pathways that typically repress TAZ or YAP would repress TAZ-CAMTA1 and inhibit EHE cell viability. TAZ-CAMTA1 lacks a critical LATS phosphorylation site through which the Hippo pathway promotes the proteasomal degradation of TAZ [125]. Although TAZ-CAMTA1 retains another critical serine residue through which LATS promotes the cytoplasmic sequestration of full-length TAZ, early studies in TAZ-CAMTA1-expressing cells suggested that TAZ-CAMTA1 is resistant to the regulation by LATS [8,9]. This may suggest that the strong nuclear localization sequence contributed by the CAMTA1 portion of the fusion [9] overrides the inhibitory phosphorylation by LATS and drives TAZ-CAMTA1 to the nucleus. Nevertheless, the TAZ portion of TAZ-CAMAT1 retains other residues subject to Hippo-independent regulation [126].

In NIH3T3 and HEK293 cells it appears that the activation of the pathways that regulate some of these residues can indeed repress the transcriptional activity of TAZ-CAMTA1. In addition to AMPK and GSK3β, we also found that PTPN14, Gelsolin, and Cofilin could each repress TAZ-CAMTA1. Gelsolin and Cofilin repress YAP and TAZ through the disruption of the F-actin cytoskeleton [40], while PTPN14 can regulate YAP through multiple mechanisms [26,38,39,41]. While this suggests that the TAZ-CAMTA1 protein is still subject to negative regulation via its N-terminus, whether GSK3β, PTPN14, Gelsolin, or Cofilin can regulate TAZ-CAMTA1 in EHE cells needs to be further explored given our finding that AMPK repressed TAZ-CAMTA1-TEAD in NIH3T3 and HEK293 but not in EHE cells. This highlights the importance of using EHE model systems to study TAZ-CAMTA1 regulation and function.

## 5. Conclusions

In conclusion, our study highlights AMPK as a promising therapeutic target for EHE. We demonstrate that AMPK activation suppresses mTOR signaling to slow EHE cell growth. This not only reinforces the growing interest in mTOR inhibitors like Sirolimus for EHE treatment but also introduces AMPK activation as an alternative strategy to target the mTOR pathway. However, the potential of AMPK is not without its caveats. While AMPK activation clearly limits EHE cell proliferation in vitro, we were unable to determine its effects in vivo due to limitations with our murine allograft models, which fail to develop aggressive EHE tumors. It will also be important to confirm these findings in human EHE cells lines, once available. Therapeutically, AMPK activation still faces hurdles. AICAR, though effective as a research compound, lacks clinical viability due to its short half-life and poor bioavailability [127]. MK8722 marked a significant advance with its improved potency, selectivity, and pharmacokinetics in vivo [47,48] but is hindered by side effects like cardiac hypertrophy [48]. We also tested metformin, a widely used and well-tolerated AMPK activator. Still, the continued development of new AMPK-activating compounds [128,129] offers hope that AMPK could become a powerful tool in the fight against EHE in the future.

In parallel, our research uncovers new insights into the regulation of the TAZ-CAMTA1 fusion protein in EHE. Notably, we discovered that TAZ-CAMTA1 remains susceptible to inhibition via its N-terminal region, highlighting a novel and potentially powerful therapeutic avenue to disrupt this oncogenic driver. We also show that the TAZ-CAMTA1-TEAD activity must be carefully balanced as excessive or insufficient activity both proved detrimental to EHE cells. While this reinforces the concept that the effective inhibition of TAZ-CAMTA1-TEAD could suppress EHE cell growth, our findings also suggest a more complex picture where, in some contexts, TAZ-CAMTA1-TEAD activity may be so elevated that it paradoxically suppresses EHE growth. In such cases, partial inhibition might inadvertently relieve this suppression and enhance tumor growth. This may shed light on results that emerge from ongoing early-phase clinical trials testing TEAD inhibitors in EHE patients. Based on our findings, it may also be interesting to investigate whether other YAP and TAZ fusion proteins that drive human cancers [130,131,132] remain susceptible to negative regulation through their N-terminus and if this is context-dependent.

## Data Availability

No omics data were generated in this study. All other data is provided in the manuscript or Supplemental Materials.

## Supplementary Materials

The following supporting information can be downloaded at https://www.mdpi.com/article/10.3390/cancers17172889/s1.

## Abbreviations

The following abbreviations are used in this manuscript:

AMP	Adenosine Monophosphate
AMPK	AMP-Activated Protein Kinase
AICAR	5-Aminoimidazole-4-Carboxyamide Ribonucleoside
ATP	Adenosine Triphosphate
CAMTA1	Calmodulin-Binding Transcription Activator 1
EHE	Epithelioid Hemangioendothelioma
FDA	Food and Drug Administration
GAP	GTPase-Activating Protein
GDP	Guanosine Diphosphate
GTP	Guanosine Triphosphate
GSK3b	Glycogen Synthase Kinase-3 Beta
LATS	Large Tumor Suppressor Kinase
mTOR	Mechanistic Target of Rapamycin
PTPN14	Protein Tyrosine Phosphatase Non-Receptor Type 14
PDX	Patient-Derived Xenograft
Rheb	Ras Homolog Enriched In Brain
TAZ	Transcriptional Co-activator with PDZ-Binding Motif
TC	TAZ-CAMTA1
TEAD	TEA Domain
TFE3	Transcription Factor E3
TSC2	Tuberous Sclerosis 2 Protein
YAP	Yes-Associated Protein 1
*WWTR1*	WW Domain-Containing Transcription Regulator Protein 1
