# Supplementary material for: AMPK Signaling Regulates Epithelioid Hemangioendothelioma Cell Growth
Source: Cancers (Basel). 2025 Sep 2;17(17):2889. doi: 10.3390/cancers17172889 (PMC12427514; doi:10.3390/cancers17172889)
Supplement: Supplementary file 1 [file cancers-17-02889-s001.zip › Supplemental Figure Legends.docx]

**Supplemental Figure Legends**

**Figure S1.** EHE cells are sensitive to TEAD inhibition. EHE6 (**A**) and EHE17 (**B-D**) cells were treated with the indicated doses of the TEAD inhibitor MGH-CP1 for 24 h (**A,B**) or every day for 4 days (**C,D**) and assayed for TEAD transcriptional activity (**A,B**), or for cell viability using a CCK8 assay (**C,D**). (**A,B,D**) The plots show the mean ± SD and each group was normalized to untreated cells (represented by the dotted line at Y=1). (**A,B**) n=3 independent experiments where 2 replicate wells read in duplicate were averaged; ****p ≤ 0.0001 by One-way ANOVA with Dunnett’s post-hoc test comparing each group to vehicle control. (**C**) For each group, the absorbance at each timepoint was normalized to the absorbance of untreated cells at the 24-hour timepoint for n=1 experiment where 4 wells were averaged. (**D**) n=4 independent experiments where 4 replicate wells were averaged; ****p ≤ 0.0001 by unpaired t-test.

**Figure S2.** TAZ-CAMTA1 promotes TEAD transcriptional activity in NIH3T3 and HEK293 cells. (**A-C**) NIH3T3 cells were stably transduced with a control empty vector (EV) or increasing amounts of TAZ-CAMTA1 retroviral supernatant, selected with neomycin, and then assayed by Western Blot (**A**) or for TEAD transcriptional activity (**B,C**). (**D,E**) HEK293 cells were stably transduced with EV, TAZ-CAMTA1, or mutants of TAZ-CAMTA1 with either a WW-domain deletion (∆WW), or that can’t bind to the TEAD transcription factors (S51A) and then assayed by Western blot (**D**) or for TEAD transcriptional activity (**E**). (**A,D**) Representative Western blots. (**B,C,E**) The plots show the mean ± SD and each group was normalized to uninfected parental cells (represented by the dotted line at Y=1). (**B**) n=4 wells where duplicate reads were averaged from 2 independent experiments; ns = not significant, ***p ≤ 0.001, ****p ≤ 0.0001 by unpaired t-test. (**C**) n=3 independent experiments where 2 wells read in duplicate were averaged; *p≤0.05 by unpaired t-test. (**E**) n=4 independent experiments where 2 wells read in duplicate were averaged; ns = not significant, ****p ≤ 0.0001 by One-way ANOVA with Dunnett’s post-hoc test comparing each group to EV control.

**Figure S3**. Identification of regulators of TAZ-CAMTA1-TEAD transcriptional activity. (**A**) The table lists known regulators of the N-terminus of YAP or TAZ with corresponding references. (**B**) Schematic showing how candidate proteins are predicted to regulate the N-terminus of TAZ-CAMTA1. (**C**) NIH3T3 cells expressing TAZ-CAMTA1 (TC) were co-transfected with TEAD transcriptional reporter constructs and either a control empty vector (EV) or an expression plasmid encoding a candidate repressor and then assayed for TEAD transcriptional activity. (**D**) Putative repressors were re-screened using the same approach in HEK293 cells expressing TAZ-CAMTA1 (TC). All plots show the mean ± SD and each group was normalized to cells transfected only with reporter constructs (represented by the dotted line at Y=1). n=4 independent experiments where 2 wells read in duplicate were averaged; ns = not significant, ****p ≤ 0.0001, ***p ≤ 0.001, **p ≤ 0.01, *p ≤ 0.05 by One-way ANOVA with Dunnett’s post hoc test comparing each group to TAZ-CAMTA1 cells transfected with EV control.

**Figure S4.** AMPK activation inhibits EHE cell viability. EHE17 cells were treated with the indicated doses of AICAR (**A-C**) or MK8722 (**D-F**) every day for 4 days (**A,C,D,F**) or for 24 h (**B,E**) and then assayed for cell viability using a CCK8 assay (**A,C,D,F**) or by Western Blot (**B,E**). (**A,D**) Each group was normalized to the absorbance of untreated cells at the 24-hour timepoint; n=1 independent experiment where 4 wells were averaged. (**B,E**) Representative Western blots with quantification of phosphorylated over total AMPK and ACC shown. (**C,F**) The plots show the mean ± SD and each group was normalized to untreated cells (represented by dotted lines at Y=1 on graphs), n=3 independent experiments where 4 replicate wells were averaged; ***p ≤ 0.001 by unpaired t-test.

**Figure S5.** MK8722 reduces EHE cell proliferation but does not induce apoptosis. EHE6 cells were treated with MK8722 for 96 h and assayed by immunofluorescence for Ki67 **(A,B)** or by a SPiDER-βGal kit for β-Galactosidase (β-Gal) **(C)**. **(A)** Representative fields of view showing Dapi (blue) and Ki67 (green) positive nuclei (with quantification in (**B**). (**D-F**) EHE6 cells were treated with MK8722 for 24 h and assayed by Western blot for changes in total and cleaved Caspase-3 and PARP. (**D**) Representative image of Western blots with quantification. **(E,F)** Band intensity was quantified and normalized to β-actin. For all graphs, data represent mean ± SD and each group was normalized to untreated cells (represented by dotted lines at Y=1 on graphs). (**A-C**) n=3 independent experiments where 2 (**A-B**) or 4 (**C**) replicate wells were averaged**p ≤ 0.01, ns = not significant by unpaired t-test. (**E,F**) n=3 independent experiments; ns = not significant by unpaired t-test.

**Figure S6.** AMPK activation increases TAZ-CAMTA1 and decreases YAP protein levels. EHE17 (**A,B**) and EHE6 (**C-G**) cells were treated for 24 h with AICAR (**A**) or MK8722 (**B-G**) and assayed for TEAD transcriptional activity (**A,B**), by Western blot (**C-E**), or by qPCR (**F,G**). The plots show the mean ± SD. (**A,B**) Each group was normalized to untreated cells (represented by dotted lines at Y=1 on graphs), n=3 independent experiments where 2 wells read in duplicate were averaged; ns = not significant, ****p ≤ 0.0001, ***p ≤ 0.001, **p ≤ 0.01 by One-way ANOVA with Dunnett’s Post-hoc test comparing each group to vehicle treated cells. (**C**) Representative Western blots with band intensity quantified and normalized to GAPDH (**D,E**). n=4 independent experiments where each group was normalized to vehicle treated cells (represented by dotted lines at Y=1 on graphs); ns = not significant, *p ≤ 0.05 by one sample t-test. (**F,G**) n=3 independent experiments where 3 technical replicates were averaged and each group was normalized to untreated cells (represented by dotted lines at Y=1 on graphs); ns = not significant by unpaired t-test.

**Figure S7.** The mTOR inhibitor rapamycin inhibits EHE cell viability. (**A-D**) EHE17 cells were treated with the mTOR inhibitor rapamycin at the indicated doses every day for 4 days (**A-C**) or for 24 h (**D**) and then cells were assayed for cell viability using a CCK8 assay (**A,B**) and by counting cell number (**C**), or by Western blot (**D**). (**A**) Each group was normalized to the absorbance of untreated cells at the 24-hour timepoint; n=1 independent experiment where 4 wells were averaged. (**B,C**) The plots show the mean ± SD and each group was normalized to untreated cells (represented by dotted lines at Y=1 on graphs), n=3 independent experiments where 4 replicate wells were averaged; ****p ≤ 0.001 by unpaired t-test. (**D**) Representative Western blots.

**Table S1. List of constructs and their sources** Existing and new vectors are listed with their source and the citation that describes them. For new constructs, the backbone and insert are listed

**Table S2. Primary antibody list.** Primary antibodies used in this study, their source, and the dilution they were use at are listed.

**Table S3. qPCR primers.**
