# Supplementary material for: AMPK Signaling Regulates Epithelioid Hemangioendothelioma Cell Growth
Source: Cancers (Basel). 2025 Sep 2;17(17):2889. doi: 10.3390/cancers17172889 (PMC12427514; doi:10.3390/cancers17172889)
Supplement: Supplementary file 1 [file cancers-17-02889-s001.zip › Supplemental Figure Legends.pdf]

Supplemental Figure Legends

Figure S1. EHE cells are sensitive to TEAD inhibition. EHE6 (**A**) and EHE17 (**B-D**) cells were treated with the indicated doses of the TEAD inhibitor MGH-CP1 for 24 h (**A,B**) or every day for 4 days (**C,D**) and assayed for TEAD transcriptional activity (**A,B**), or for cell viability using a CCK8 assay (**C,D**). (**A,B,D**) The plots show the mean ± SD and each group was normalized to untreated cells (represented by the dotted line at Y=1). (**A,B**) n=3 independent experiments where 2 replicate wells read in duplicate were averaged; ****$p \leq 0.0001$ by One-way ANOVA with Dunnett's post-hoc test comparing each group to vehicle control. (**C**) For each group, the absorbance at each timepoint was normalized to the absorbance of untreated cells at the 24-hour timepoint for n=1 experiment where 4 wells were averaged. (**D**) n=4 independent experiments where 4 replicate wells were averaged; ****$p \leq 0.0001$ by unpaired t-test.

Figure S2. TAZ-CAMTA1 promotes TEAD transcriptional activity in NIH3T3 and HEK293 cells. (**A-C**) NIH3T3 cells were stably transduced with a control empty vector (EV) or increasing amounts of TAZ-CAMTA1 retroviral supernatant, selected with neomycin, and then assayed by Western Blot (**A**) or for TEAD transcriptional activity (**B,C**). (**D,E**) HEK293 cells were stably transduced with EV, TAZ-CAMTA1, or mutants of TAZ-CAMTA1 with either a WW-domain deletion (ΔWW), or that can't bind to the TEAD transcription factors (S51A) and then assayed by Western blot (**D**) or for TEAD transcriptional activity (**E**). (**A,D**) Representative Western blots. (**B,C,E**) The plots show the mean ± SD and each group was normalized to uninfected parental cells (represented by the dotted line at Y=1). (**B**) n=4 wells where duplicate reads were averaged from 2 independent experiments; ns = not significant, ***$p \leq 0.001$, ****$p \leq 0.0001$ by unpaired t-test. (**C**) n=3 independent experiments where 2 wells read in duplicate were averaged; *$p \leq 0.05$ by unpaired t-test. (**E**) n=4 independent experiments where 2 wells read in duplicate were averaged; ns = not significant, ****$p \leq 0.0001$ by One-way ANOVA with Dunnett's post-hoc test comparing each group to EV control.

Figure S3. Identification of regulators of TAZ-CAMTA1-TEAD transcriptional activity. (**A**) The table lists known regulators of the N-terminus of YAP or TAZ with corresponding references. (**B**) Schematic showing how candidate proteins are predicted to regulate the N-terminus of TAZ-CAMTA1. (**C**) NIH3T3 cells expressing TAZ-CAMTA1 (TC) were co-transfected with TEAD transcriptional reporter constructs and either a control empty vector (EV) or an expression plasmid encoding a candidate repressor and then assayed for TEAD transcriptional activity. (**D**) Putative repressors were re-screened using the same approach in HEK293 cells expressing TAZ-CAMTA1 (TC). All plots show the mean ± SD and each group was normalized to cells transfected only with reporter constructs (represented by the dotted line at Y=1). n=4 independent experiments where 2 wells read in duplicate were averaged; ns = not significant, ****$p \leq 0.0001$, ***$p \leq 0.001$, **$p \leq 0.01$, *$p \leq 0.05$ by One-way ANOVA with Dunnett's post hoc test comparing each group to TAZ-CAMTA1 cells transfected with EV control.

Figure S4. AMPK activation inhibits EHE cell viability. EHE17 cells were treated with the indicated doses of AICAR (**A-C**) or MK8722 (**D-F**) every day for 4 days (**A,C,D,F**) or for 24 h (**B,E**) and then assayed for cell viability using a CCK8 assay (**A,C,D,F**) or by Western Blot (**B,E**). (**A,D**) Each group was normalized to the absorbance of untreated cells at the 24-hour timepoint; n=1 independent experiment where 4 wells were averaged. (**B,E**) Representative Western blots with quantification of phosphorylated over total AMPK and ACC shown. (**C,F**) The plots show the mean ± SD and each group was normalized to untreated cells (represented by dotted lines at Y=1 on graphs), n=3 independent experiments where 4 replicate wells were averaged; ***$p \leq 0.001$ by unpaired t-test.

Figure S5. MK8722 reduces EHE cell proliferation but does not induce apoptosis. EHE6 cells were treated with MK8722 for 96 h and assayed by immunofluorescence for Ki67 **(A,B)** or by a SPiDER-βGal kit for β-Galactosidase (β-Gal) **(C). (A)** Representative fields of view showing Dapi (blue) and Ki67 (green) positive nuclei (with quantification in (**B**). (**D-F**) EHE6 cells were treated with MK8722 for 24 h and assayed by Western blot for changes in total and cleaved Caspase-3 and PARP. (**D**) Representative image of Western blots with quantification. **(E,F)** Band intensity was quantified and normalized to β-actin. For all graphs, data represent mean ± SD and each group was normalized to untreated cells (represented by dotted lines at Y=1 on graphs). (**A-C**) n=3 independent experiments where 2 (**A-B**) or 4 (**C**) replicate wells were averaged**$p \leq 0.01$, ns = not significant by unpaired t-test. (**E,F**) n=3 independent experiments; ns = not significant by unpaired t-test.

Figure S6. AMPK activation increases TAZ-CAMTA1 and decreases YAP protein levels. EHE17 (**A,B**) and EHE6 (**C-G**) cells were treated for 24 h with AICAR (**A**) or MK8722 (**B-G**) and assayed for TEAD transcriptional activity (**A,B**), by Western blot (**C-E**), or by qPCR (**F,G**). The plots show the mean ± SD. (**A,B**) Each group was normalized to untreated cells

(represented by dotted lines at Y=1 on graphs), n=3 independent experiments where 2 wells read in duplicate were averaged; ns = not significant, ****$p \leq 0.0001$, ***$p \leq 0.001$, **$p \leq 0.01$ by One-way ANOVA with Dunnett's Post-hoc test comparing each group to vehicle treated cells. (**C**) Representative Western blots with band intensity quantified and normalized to GAPDH (**D,E**). n=4 independent experiments where each group was normalized to vehicle treated cells (represented by dotted lines at Y=1 on graphs); ns = not significant, *$p \leq 0.05$ by one sample t-test. (**F,G**) n=3 independent experiments where 3 technical replicates were averaged and each group was normalized to untreated cells (represented by dotted lines at Y=1 on graphs); ns = not significant by unpaired t-test.

Figure S7. The mTOR inhibitor rapamycin inhibits EHE cell viability. (**A-D**) EHE17 cells were treated with the mTOR inhibitor rapamycin at the indicated doses every day for 4 days (**A-C**) or for 24 h (**D**) and then cells were assayed for cell viability using a CCK8 assay (**A,B**) and by counting cell number (**C**), or by Western blot (**D**). (**A**) Each group was normalized to the absorbance of untreated cells at the 24-hour timepoint; n=1 independent experiment where 4 wells were averaged. (**B,C**) The plots show the mean ± SD and each group was normalized to untreated cells (represented by dotted lines at Y=1 on graphs), n=3 independent experiments where 4 replicate wells were averaged; ****$p \leq 0.001$ by unpaired t-test. (**D**) Representative Western blots.

Table S1. List of constructs and their sources Existing and new vectors are listed with their source and the citation that describes them. For new constructs, the backbone and insert are listed

Table S2. Primary antibody list. Primary antibodies used in this study, their source, and the dilution they were use at are listed.

Table S3. qPCR primers.
