# Supplementary material for: AMPK Signaling Regulates Epithelioid Hemangioendothelioma Cell Growth
Source: Cancers (Basel). 2025 Sep 2;17(17):2889. doi: 10.3390/cancers17172889 (PMC12427514; doi:10.3390/cancers17172889)
Supplement: Supplementary file 1 [file cancers-17-02889-s001.zip › Supplemental Table 1.pdf]

Table S1

Existing/Purchased/Gift Plasmids

Plasmid Name	Source of Plasmid	Reference (if applicable)
VSVG	Lamar Lab	Lamar et al. 2019
gag/pol	Addgene #14887	Reya et al. 2003
psPAX2	Addgene #12260	Unpublished
pLenti-PGK-Blast-Renilla Luciferase	Addgene #74444	Toyama et al. 2016
pLenti-8xGTIIC-Firefly Luciferase-Puro	Rubin Lab	Seavey et al. 2023
pBABE-Neo-2xFlag-TAZ-CAMTA1	Rubin Lab	Tanas et al. 2016
pQ2aB	Addgene #124887	Unpublished
pGL3-5xMCAT(SV)-49	Iain Farrance	Mahoney et al. 2005
PRL-TK	Promega, Cat# E2241	Unpublished
pECE-HA-AMPKα1 WT	Addgene #69504	Schaffer et al. 2015
pECE-HA-AMPKα2 WT	Addgene #31654	Banko et al. 2011
pCDNA3-V5-PTPN14-wild type	Addgene #61003	Wilson et al. 2014
pEGFP-N1-human cofilin s3a	Addgene #50860	Garvalov et al. 2007
pEGFP-N1-human cofilin WT	Addgene #50859	Garvalov et al. 2007
pCMV-DeAct-GS1	Addgene #89445	Harterink et al. 2017
HA GSK3 beta S9A pcDNA3	Addgene #14754	Stambolic and Woodgett 1994
pCDNA3-HA-Angiomotin p130	Addgene #32821	Zhao et al. 2011
MSCV-NF2A-IRES-Puro	Hynes Lab	Unpublished
MSCV-NF2B-IRES-Puro	Hynes Lab	Unpublished

New Vectors

Vector	Source Backbone	Source Insert
pQ2aB-2xFlag-TAZ-CAMTA1	pBABE-Neo-2xFlag-TAZ-CAMTA1	pQ2aB
