# Supplementary material for: AMPK Signaling Regulates Epithelioid Hemangioendothelioma Cell Growth
Source: Cancers (Basel). 2025 Sep 2;17(17):2889. doi: 10.3390/cancers17172889 (PMC12427514; doi:10.3390/cancers17172889)
Supplement: Supplementary file 1 [file cancers-17-02889-s001.zip › Supplemental Table 2.pdf]

Table S2

Antibody Information	Antibody Catalog Number	Use	Dilution
**TAZ** (E8E9G) Rabbit Monoclonal Antibody	Cell Signaling, Cat# 83669	Western Blot	1:1000
**YAP** XP (D8H1X) Rabbit Monoclonal Antibody	Cell Signaling, Cat# 14074	Western Blot	1:1000
**β-Actin** (13E5) Rabbit Monoclonal Antibody	Cell Signaling, Cat# 4970	Western Blot	1:1000
**GAPDH** (14C10) Rabbit Monoclonal Antibody	Cell Signaling, Cat# 2118	Western Blot	1:1000
**Phospho-AMPKα (Thr172)** (40H9) Rabbit Monoclonal Antibody	Cell Signaling, Cat# 2535	Western Blot	1:1000
**AMPKα** (D5A2) Rabbit Monoclonal Antibody	Cell Signaling, Cat# 5831	Western Blot	1:1000
**Acetyl-CoA Carboxylase (ACC)** (C83B10) Rabbit Monoclonal Antibody	Cell Signaling, Cat# 3676	Western Blot	1:1000
**Phospho-Acetyl-CoA Carboxylase (Ser79)** (D7D11) Rabbit Monoclonal Antibody	Cell Signaling, Cat# 11818	Western Blot	1:1000
**Cleaved PARP (Asp214)** (D64E10) XP Rabbit Monoclonal Antibody	Cell Signaling, Cat# 5625	Western Blot	1:1000
**PARP** Rabbit Polyclonal Antibody	Cell Signaling, Cat# 9542	Western Blot	1:1000
**Cleaved Caspase-3 (Asp175)** (5A1E) Rabbit Monoclonal Antibody	Cell Signaling, Cat# 9664	Western Blot	1:1000
**Caspase-3** (8G10) Rabbit Monoclonal Antibody	Cell Signaling, Cat# 9665	Western Blot	1:1000
**PCNA** Mouse Monoclonal Antibody (PC10 (3F81))	Thermo Fisher, Cat# 14-9910-80	Western Blot	1:500
Goat anti-Rabbit IgG (H+L) 2º Ab, Horseradish Peroxidase conjugated	Thermo Fisher, Cat# 31460	Western Blot	1:5000
Goat anti-Mouse IgG (H+L) 2º Ab, Horseradish Peroxidase conjugated	Thermo Fisher, Cat# 32430	Western Blot	1:5000
**Ki67** (SP6) Rabbit Monoclonal Antibody	Thermo Fisher, Cat# MA5-14520	Immunocytochemistry/Immunofluorescence	1:250
Goat anti-Rabbit IgG (H+L) Cross-Adsorbed Secondary Antibody, Alexa Fluor™ 488	Thermo Fisher, Cat# A-11008	Immunocytochemistry/Immunofluorescence	1:500
