# Supplementary material for: AMPK Signaling Regulates Epithelioid Hemangioendothelioma Cell Growth
Source: Cancers (Basel). 2025 Sep 2;17(17):2889. doi: 10.3390/cancers17172889 (PMC12427514; doi:10.3390/cancers17172889)
Supplement: Supplementary file 1 [file cancers-17-02889-s001.zip › Supplemental Table 3.pdf]

Table S3

Gene	Forward Primer (5' --> 3')	Reverse Primer (5' --> 3')
Taz-Camta1	CCCAGGAAGGTGATGAATCAG	TGAGATGATGCGGTGTTTG
Yap	GATGGAGGGACTCCGAATGC	GGCTGATGGTGTCTCCTGTAT
Gapdh	CTTTGTCAAGCTCATTTCCTGG	TCTTGCTCAGTGTCCTTGC
