# Supplementary figures and images for: AMPK Signaling Regulates Epithelioid Hemangioendothelioma Cell Growth
Source: Cancers (Basel). 2025 Sep 2;17(17):2889. doi: 10.3390/cancers17172889 (PMC12427514; doi:10.3390/cancers17172889)

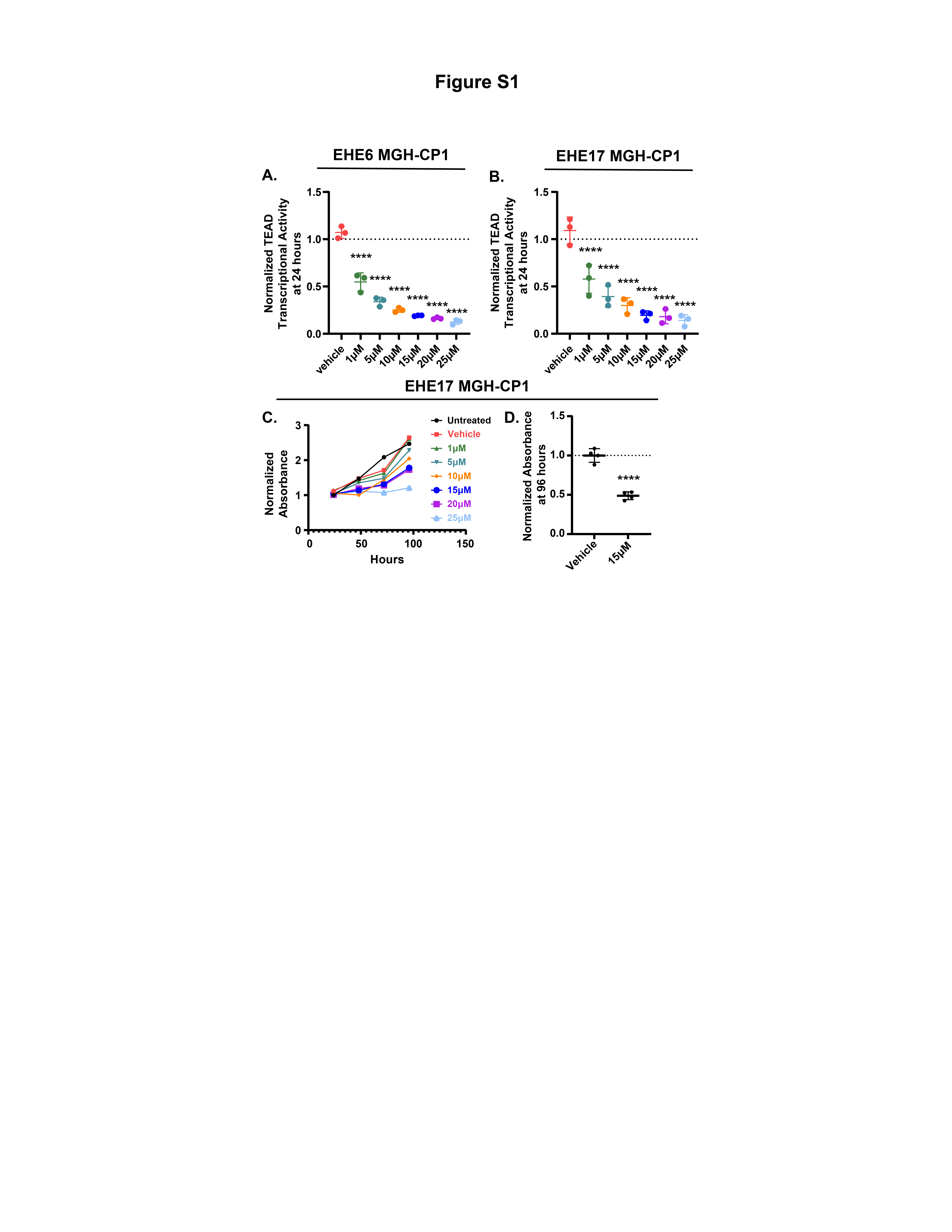

Supplement: Supplementary file 1 [file cancers-17-02889-s001.zip › Figure S1.jpg]

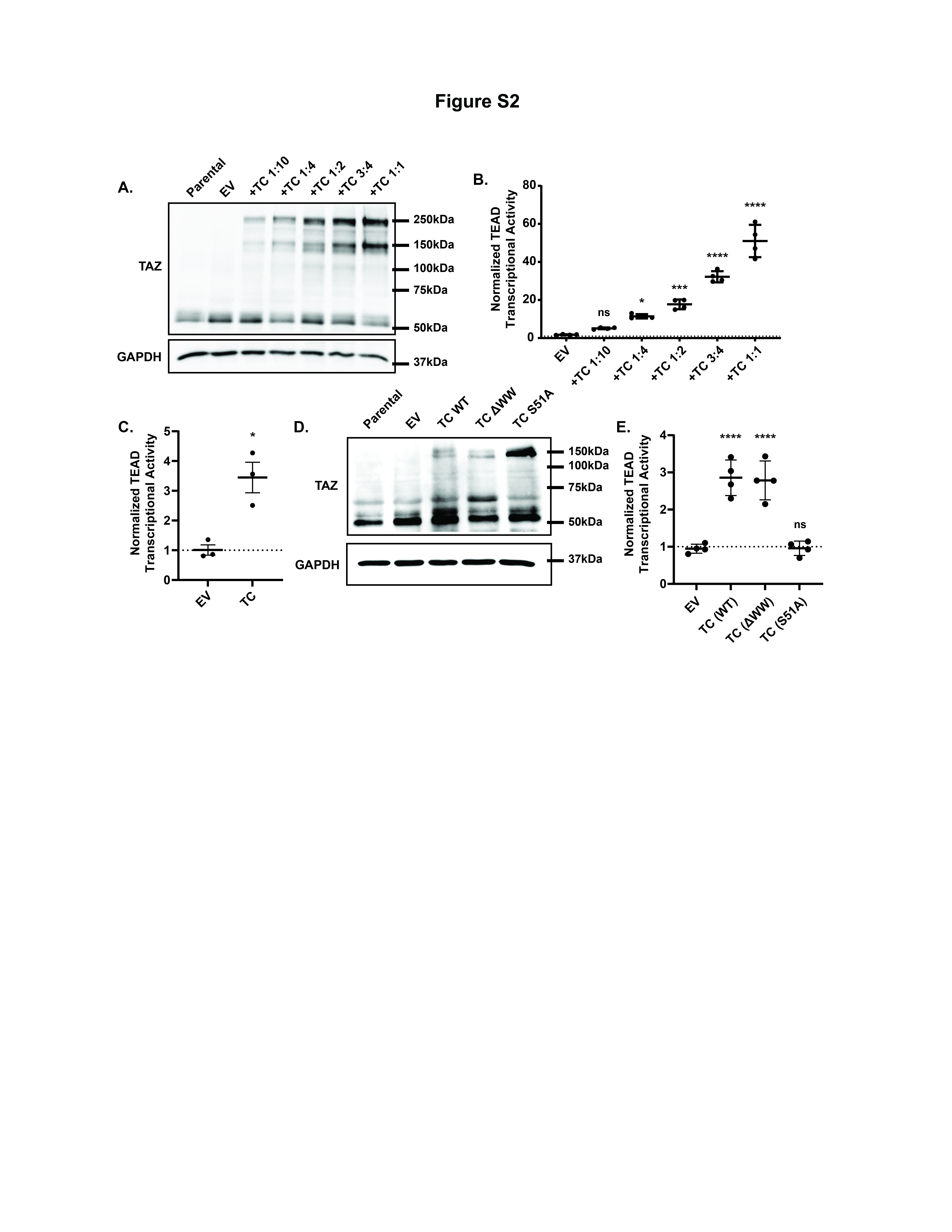

Supplement: Supplementary file 1 [file cancers-17-02889-s001.zip › Figure S2.jpg]

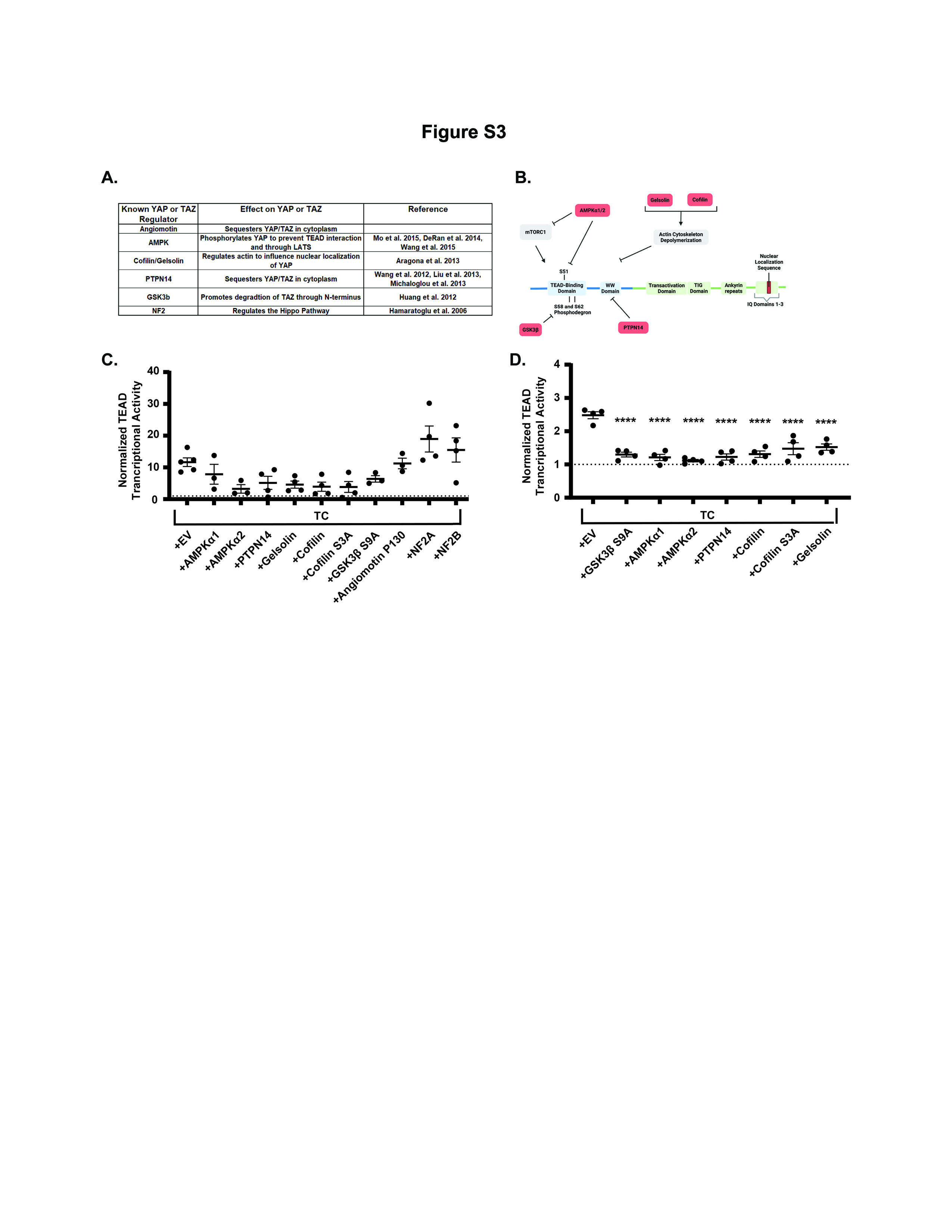

Supplement: Supplementary file 1 [file cancers-17-02889-s001.zip › Figure S3.jpg]

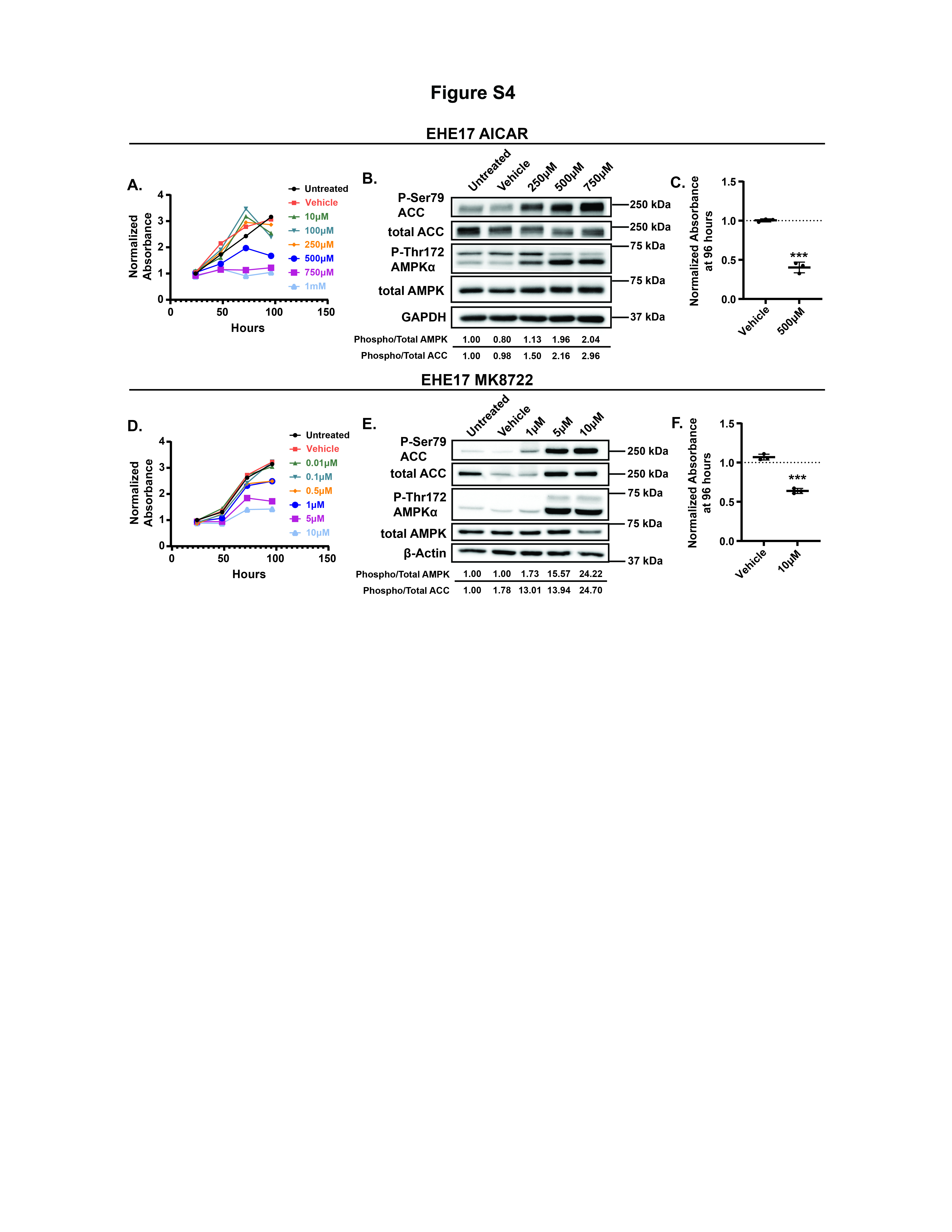

Supplement: Supplementary file 1 [file cancers-17-02889-s001.zip › Figure S4.jpg]

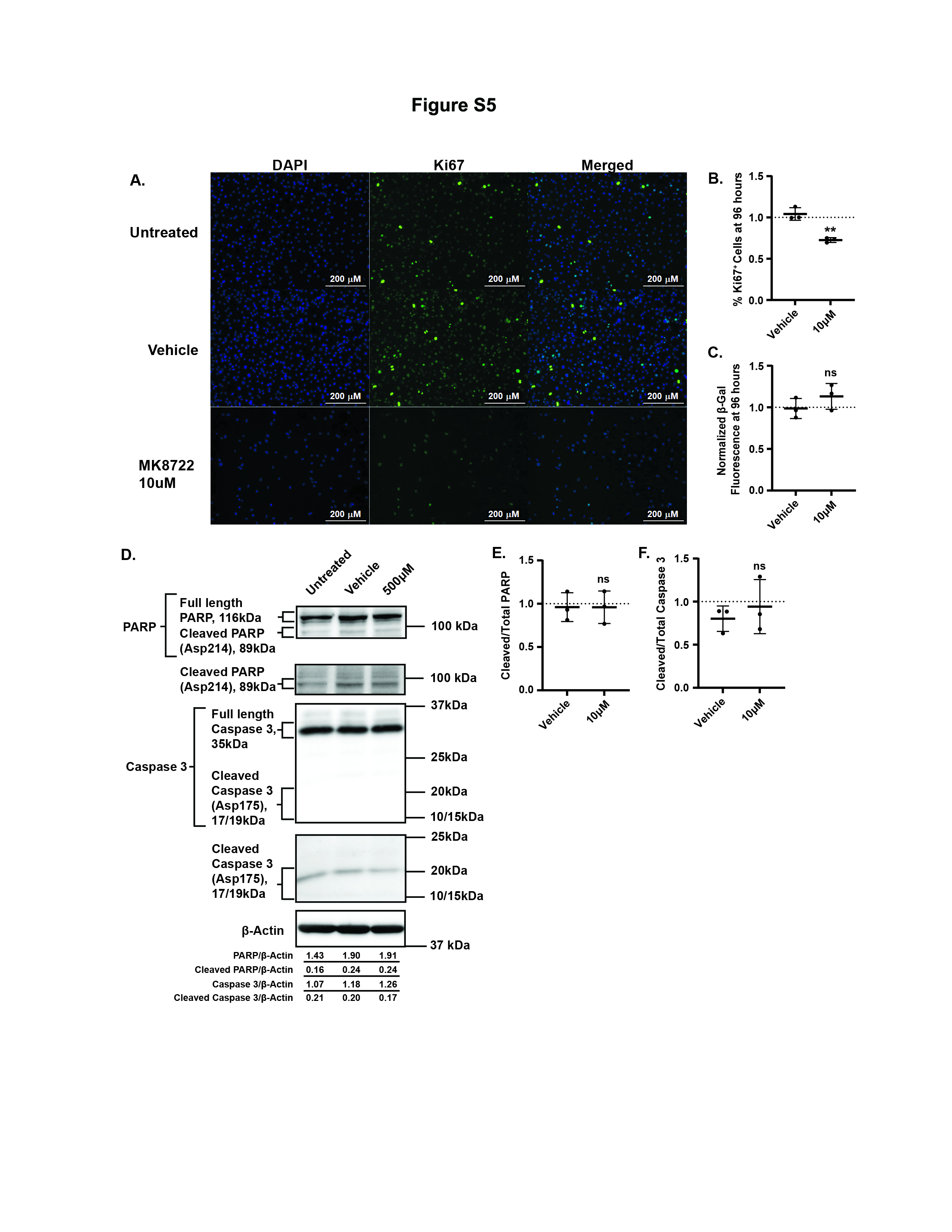

Supplement: Supplementary file 1 [file cancers-17-02889-s001.zip › Figure S5.jpg]

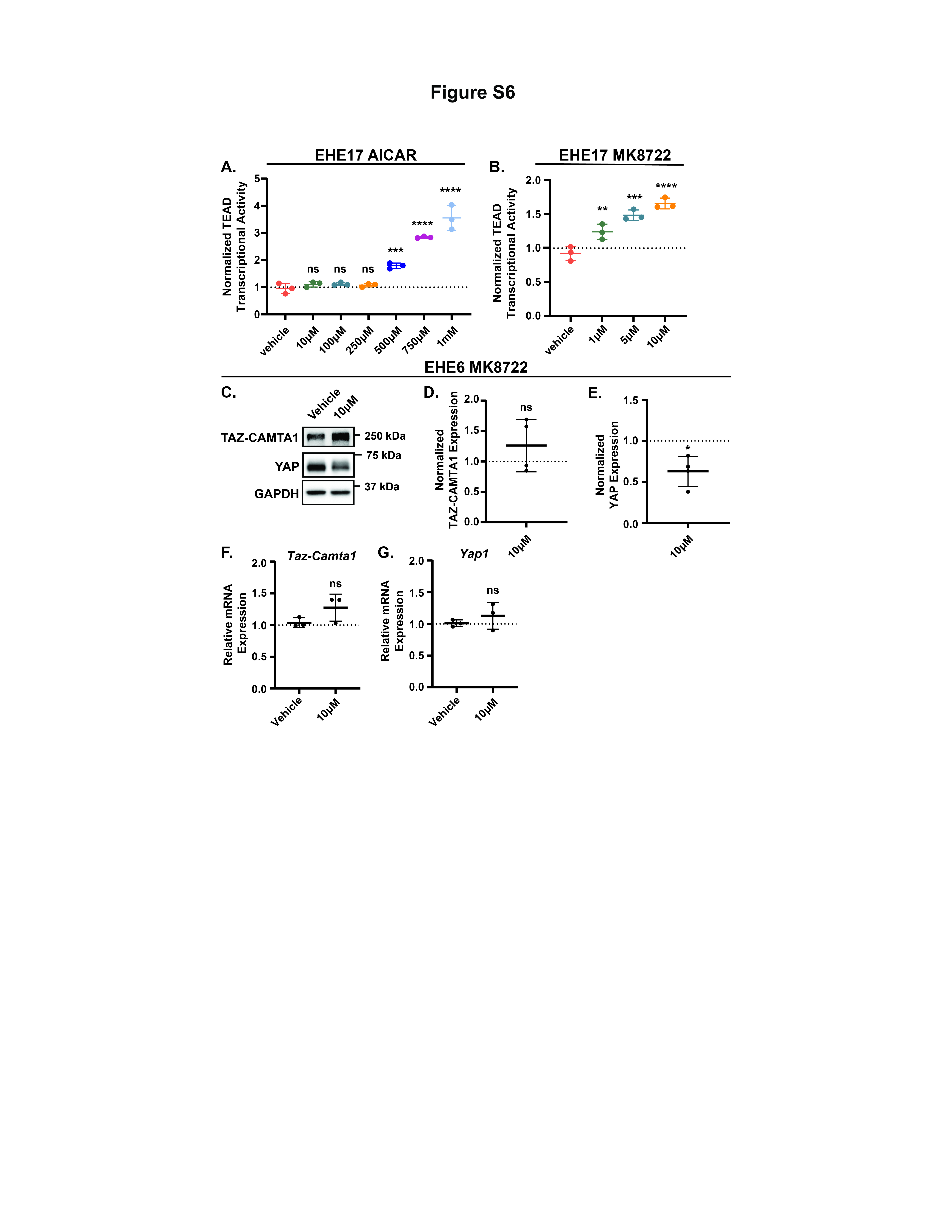

Supplement: Supplementary file 1 [file cancers-17-02889-s001.zip › Figure S6.jpg]

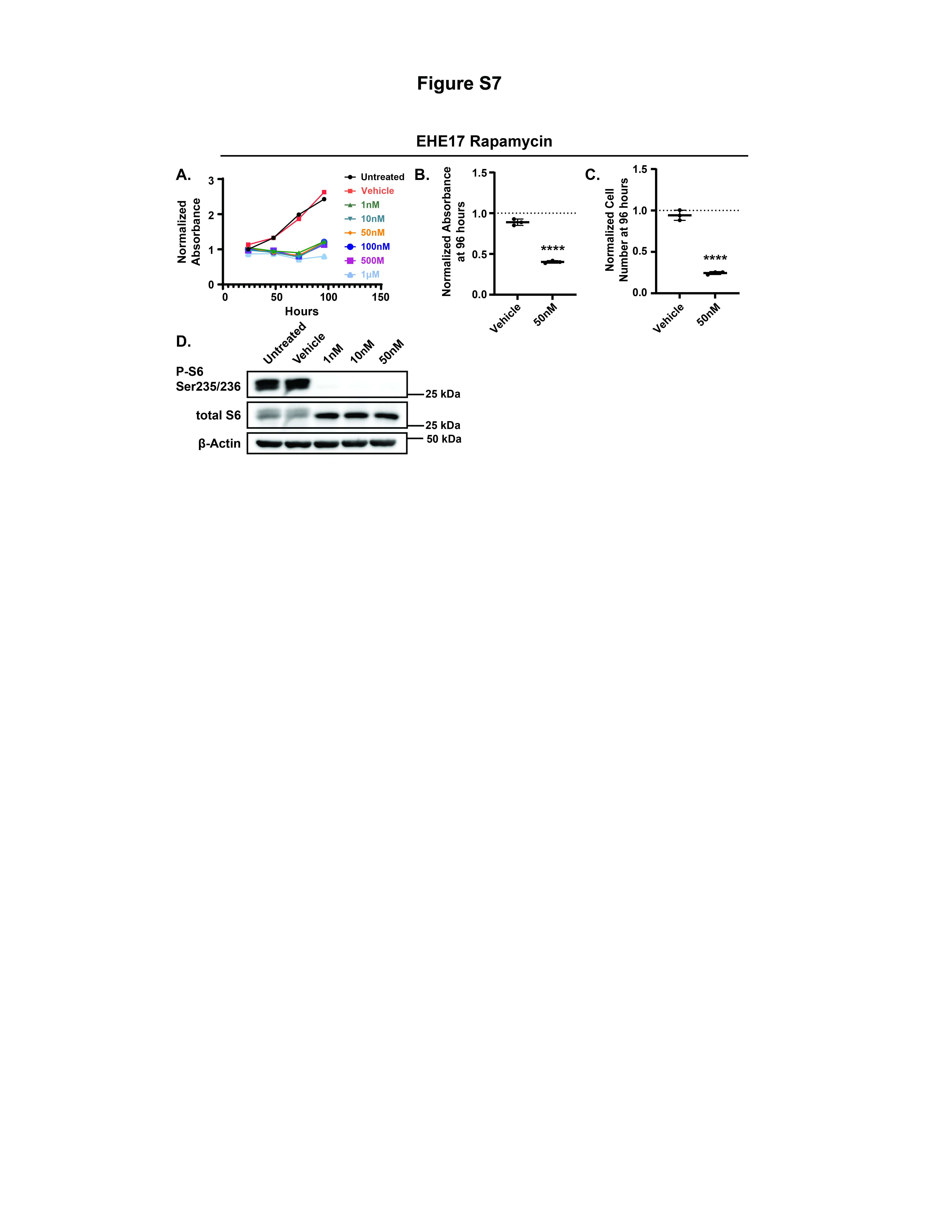

Supplement: Supplementary file 1 [file cancers-17-02889-s001.zip › Figure S7.jpg]
